# Chemistry and Biology of Bioactive Glycolipids of Marine Origin

**DOI:** 10.3390/md16090294

**Published:** 2018-08-22

**Authors:** Iván Cheng-Sánchez, Francisco Sarabia

**Affiliations:** Department of Organic Chemistry, Faculty of Sciences, University of Málaga, Campus de Teatinos s/n, 29071 Málaga, Spain

**Keywords:** glycolipids, glycosphingolipids, glycoglycerolipids, natural products, total synthesis

## Abstract

Glycolipids represent a broad class of natural products structurally featured by a glycosidic fragment linked to a lipidic molecule. Despite the large structural variety of these glycoconjugates, they can be classified into three main groups, i.e., glycosphingolipids, glycoglycerolipids, and atypical glycolipids. In the particular case of glycolipids derived from marine sources, an impressive variety in their structural features and biological properties is observed, thus making them prime targets for chemical synthesis. In the present review, we explore the chemistry and biology of this class of compounds.

## 1. Introduction

**Figure 1:**
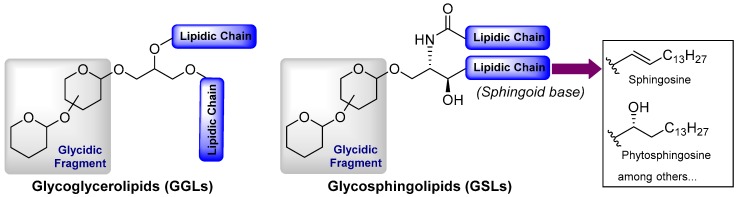
General structural feature of glycolipids.

Glycolipids represent a broad class of biologically active natural products with a wide variety of molecular structures and biological functions, many of which are essential for life [1,2]. Despite their extensive structural diversity, glycolipids can be classified by the nature of the lipidic fragment. According to this classification, these glycoconjugates are divided into three main groups, i.e., glycoglycerolipids, glycosphingolipids, and those comprising the rest of the glycolipids possessing atypical lipidic moieties. Whereas glycoglycerolipids are mainly distributed in the realm of micro-organisms and plants, glycosphingolipids are extensively found in all living beings. In fact, these molecules are found on the surface of cell membranes and, together with glycoproteins and glycosaminoglycans, which are known as glycocalyx, play critical roles for cell growth, cellular recognition, adhesion, neuronal repair, and signal transduction, which are essential for health and involved in a number of diseases such as cancer and inflammatory processes and infections [3,4,5]. Given their molecular complexity and diversity, glycosphingolipids are classified into the following subgroups: (a) neutral, which can be subdivided into the cerebrosides that contain only one uncharged sugar, the diosylceramides with two sugar units, and the neutral glycosphingolipids with more than two (up to 30) uncharged sugars; and (b) acidic, which can be subdivided into the gangliosides, characterized by the presence of one or more neuraminic acid residues, and, finally the sulfatides, which contain at least one sugar residue with a sulfate group. In any case, all of the glycosphingolipids share a common ceramide lipid unit, consisting of a long-chain amino-alcohol fragment, which can be a sphingosine or a phytosphingosine unit (sphingoid base) linked to a fatty acid via an amide bond. In the case of the glycoglycerolipids, their core structure is comprised of a 1,2-diacyl glycerol attached to a mono- or an oligosaccharide molecule, although some variations with respect to this general structure can be found, as will be described later. In contrast to the glycosphingolipids, the glycoglycerolipids are present in nature in much lower abundance, which, combined with the difficulty of their isolation from natural sources, have significantly hampered extensive and detailed biological studies. The third group consists of the atypical glycolipids that include any glycoconjugate that contains a lipidic chain not present in the previous groups (Figure 1). The stunning and limitless wealth of secondary metabolites that marine organisms provide is extensive for this class of compounds, with a myriad of glycolipid-type natural products with impressive molecular diversity and a variety of biological activities, including antitumor, antiviral, and anti-inflammatory properties. Given the biological relevance and structural complexity of these classes of compounds, a large number of reviews [6,7,8,9,10,11,12], books, and book chapters [13,14,15] have been devoted to all aspects related to their chemistry and biology. More specifically, in the field of glycolipids of marine origin, several excellent reviews have been published, especially by Barnathan et al. [16], which represents an excellent description of all the glycolipids found in marine invertebrates, as well as by Li et al. [17], which focused on the chemistry and biology of glycoglycerolipids from marine organisms. In addition, numerous reviews have been reported on very specific compounds, such as KRN7000 [18] and related glycosphingolipids [19] due to their outstanding biological activities and their potential pharmacological activity. In light of this publication landscape, this review intends to give a chemical and biological perspective of these fascinating natural products with a particular emphasis on recent contributions that were not covered in the aforementioned reviews and highlighting the importance of their synthesis given the particularly intricate requirements to obtain sufficient amounts from their natural sources. This review also provides an updated state of the art of this field that can attract the interest of chemists and biologists, revealing the potential and prospects that these compounds may provide in biology, chemistry, and biomedicine for the future.

## 2. Chemistry and Biology of Glycosphingolipids

### 2.1. Neutral Glycosphingolipids

#### 2.1.1. Cerebrosides

##### Acanthacerebrosides, Astrocerebrosides, and Asteriacerebrosides

**Figure 2:**
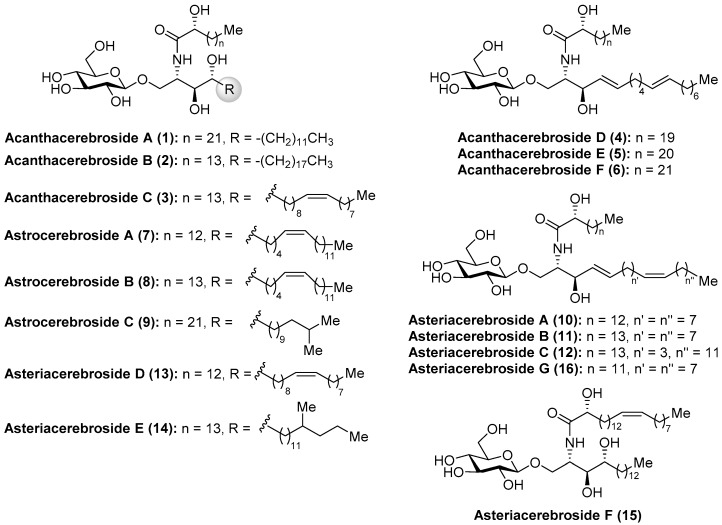
Structures of the acanthacerebrosides (**1**–**6**), astrocerebrosides (**7**–**9**), and asteriacerebrosides (**10**–**16**).

Among the wide and important family of marine glycosphingolipids containing a phytosphingosine unit, which confers the glycolipids outstanding immunostimulant properties, as will be described later, the acanthacerebrosides and astrocerebrosides represent interesting members isolated from different starfish species. It was in 1988 when Komori et al. [20] discovered the first members of the acanthacerebrosides (A–F, **1**–**6**) from the starfish *Acanthaster planci*. These compounds were isolated as a mixture, which hindered a complete structural elucidation and biological evaluations. However, subsequent purification efforts by the same group led to the isolation of one of their members, acanthacerebroside B (**2**), which could be isolated as a pure compound from the starfish *Asterina pectinifera* and allowed full structural characterization by a complete NMR spectroscopic analysis. In the same year, these authors described the first total synthesis of acanthacerebroside A (**1**), establishing unambiguously the structure and absolute configuration of this new family of cerebrosides [21]. In addition, the investigations of Komori et al. [22] with the starfish *Astropecten latespinosus* led to the discovery of three new related cerebrosides, which were named astrocerebrosides A (**7**), B (**8**), and C (**9**). Later, in 1991, the study of the starfish *Asterias amurensis versicolor* allowed Komori´s group to isolate new cerebrosides: asteriacerebrosides AߝF (**10**–**15**) [23]. Ten years later, Ishii et al. [24] discovered a new member of this class, asteriacerebroside G (**16**), from the starfish *Asterias amurensis* (Figure 2). 

**Scheme 1:**
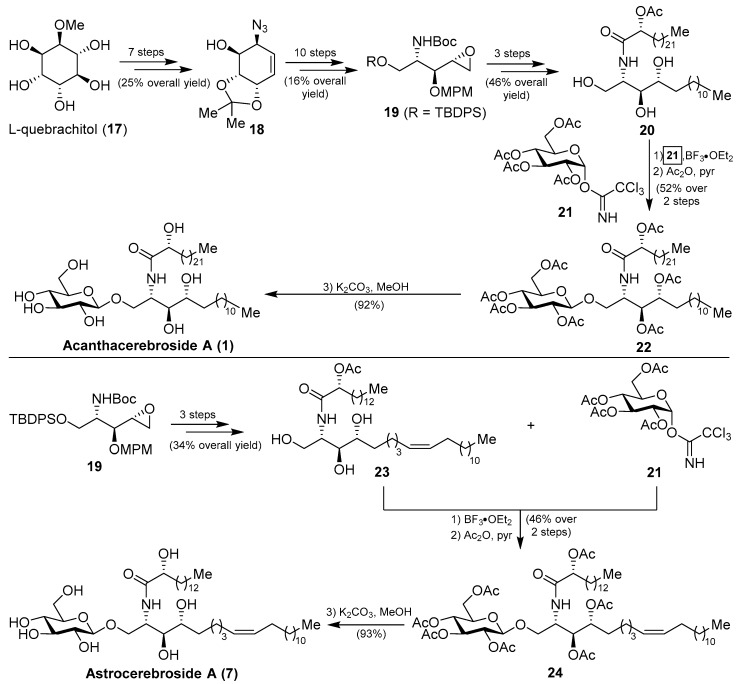
Total syntheses of acanthacerebroside A (**1**) and astrocerebroside A (**7**).

More recently, from the same starfish (*A. amurensis*), Kim et al. identified new members of related asteriacerebrosides, whose structures were established by mass spectrometric techniques [25]. After the synthesis of acanthacerebroside A (**1**) by Komori in 1998, the Chida group [26] reported a new synthesis for acanthacerebroside A (**1**) and astrocerebroside A (**7**), in which the preparation of the phytosphingosine moiety was achieved using the commercially available L-quebrachitol (**17**) (Scheme 1). Accordingly, L-quebrachitol (**17**) was transformed into the azide **18** in seven steps, which was directed towards the phytosphingosine fragment **20**, contained in acanthacerebroside A (**1**). The coupling of **20** with the donor trichloroacetimidate **21** via a glycosylation reaction mediated by BF_3_^.^OEt_2_ provided the corresponding acanthacerebroside A derivative, which was derivatized to the peracetylated **22** to facilitate purification. After removing the acetate protecting groups, natural acanthacerebroside A (**1**) was obtained. In a similar manner, astrocerebroside A (**7**) was efficiently prepared from the phytosphingosine **23**, synthesized from the common epoxide **19**, and the same glycosyl donor (**21**). From a biological standpoint, surprisingly only the asteriacerebrosides were evaluated, showing that asteriacerebrosides A (**10**), B (**11**), and G (**16**) displayed growth-promoting activity against the plant *Brassica campestris*. This was the first report of a promotive plant-growth activity of this class of compounds.

##### Agelasphins

**Figure 3:**
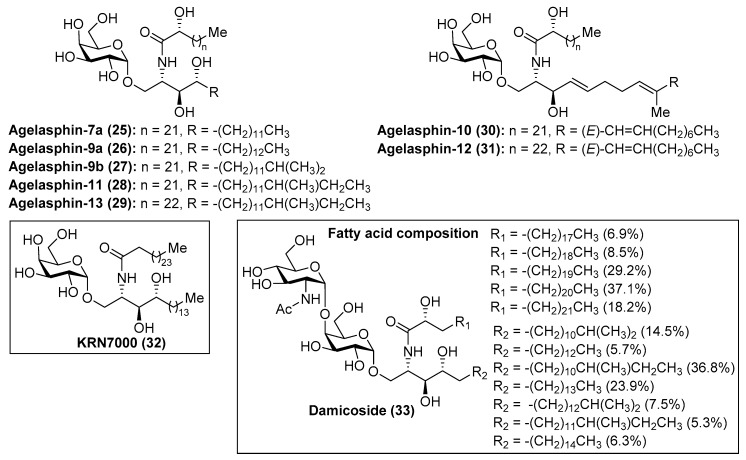
Structures of the agelasphins (**25**–**31**), KRN7000 (**32**), and damicoside (**33**).

Within the family of phytosphingosine-containing cerebrosides of marine origin, the agelasphins (**25**–**31**) (Figure 3) occupy a privileged position by virtue of their striking and promising biological properties. The presence of a galactosyl fragment instead of a glucosyl moiety confers upon them unexpected and intriguing biological properties. After the isolation of the first members of this family by Natori et al. in 1993 [27] from the marine sponge *Agelas mauritianus*, the agelasphins were rapidly recognized as antitumor compounds with weak toxicity, which elicited a great interest in chemical and biological circles. Despite these antitumoral properties, the agelasphins did not exhibit cytotoxicity against B16 melanoma cells at 20 µg/mL, which led Natori, Koezuka, et al. [28] to undertake further biological studies for a rational explanation of these intriguing properties. These studies revealed that these compounds, for example agelasphin-11 (**28**), were capable of stimulating the immune system via activation of NK cells, which explained not only their potent antitumor activity but also their immunostimulatory property. These outstanding biological findings encouraged the Koezuka’s group to achieve an extensive structure–activity relationship (SAR) study by preparation of a library of their analogues and subsequent antitumor activity evaluation from which the well-known analogue KRN7000 (**32**) was discovered [29]. At a molecular level, the activation of the immune system exerted by the agelasphins occurs because these compounds are potent ligands of the MHC class I-like CD1d protein, present on the surface of the antigen presenting cells (APCs), and, as a consequence of this potent interaction, an overwhelming response by the immune system is triggered. Thus, the invariant natural killer T cells (*i*NKT cells) are initially activated by producing high levels of cytokines, which, in turn, activate other antitumor effector cells, resulting in a strong immunological response by the organism against tumor cells. These important findings rapidly propelled KRN7000 (**32**) as a novel and promising anticancer agent, which recently entered into phase I clinical trials against various types of cancers [30]. As a consequence of its intriguing biological properties, KRN7000 has elicited widespread interest and excitement in both the biological and chemical fields. Indeed, an indication of this great interest is a flurry of activity directed toward the synthesis of a plethora of analogues for SAR studies [31,32]. Related to the agelasphins, Mangoni’s group [33] has recently identified a disaccharide derivative, named damicoside (**33**), from the marine sponge *Axinella damicornis*. This glycolipid represents the first disaccharide, structurally related to that of agelasphins, and with an immunostimulatory activity similar to the agelasphins, allowing the completion of the structure–activity relationship study of this fascinating class of compounds.

**Scheme 2:**
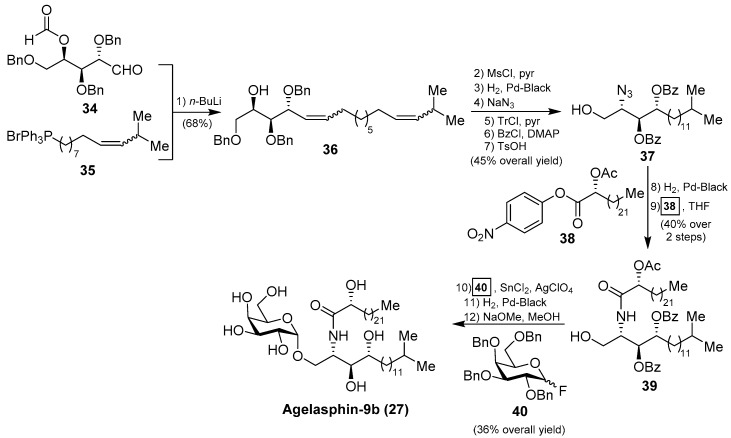
Total synthesis of agelasphin-9b (**27**).

In order to confirm the structures of the agelasphins, Natori, together with Akimoto et al. [34], achieved the first total synthesis of an agelasphin member, agelasphin-9b (**27**). Accordingly, the construction of the phytosphingosine fragment commenced with the Wittig reaction of aldehyde **34** with phosphonium salt **35** to give a mixture of isomeric alcohols **36** in 68% yield. The alcohol **36** was then converted into the azide **37** in six steps in 45% overall yield. Subsequent reduction of the azide and acetylation of the corresponding amine with *p*-nitrophenyl ester **38** afforded a protected ceramide **39** in 40% overall yield. Reaction of **39** with galactosyl fluoride **40**, under Mukaiyama’s glycosylation conditions, gave the corresponding α-galactoside in 36% yield, which was finally transformed into agelasphin-9b (**27**) after a deprotection of the protecting groups (Scheme 2). This synthesis was important not only because the authors were able to confirm the structures of these natural products, but also because the strategy for the future construction of these molecules was established and extended to the synthesis of other agelasphins and their analogues, such as KRN7000 (**32**) [18,19].

##### Axidjiferosides

**Figure 4:**
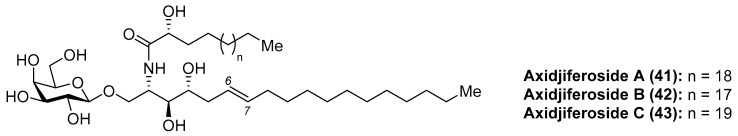
Structures of the axidjiferosides (**41**–**43**).

Barnathan et al. [35] described the isolation of axidjiferosides A–C (**41**–**43**) (Figure 4) from the marine sponge *Axinyssa djiferi*, collected from mangrove tree roots in Senegal. Interestingly, the structure of these compounds contained the unusual Δ^6^-phytosphingosine and also the unusual β-configuration of the galactopyranosyl unit. These compounds showed significant antimalarial activity, with an IC_50_ of 0.53 ± 0.2 µM against a chloroquine-resistant strain of *Plasmodium falciparum*. In addition, the axidjiferosides also showed antiplasmodial activity, with low cytotoxicity against various human cancer cell lines, and no significant antitrypanosomal and antileishmanial activities. In contrast to the α-galactosylceramide derivatives, such as the agelasphins, which displayed immunostimulating properties, the antimalarial activity exhibited by the axidjiferosides was ascribed by the authors to the β anomeric configuration present in these natural products.

##### Cerebrosides CE

**Figure 5:**
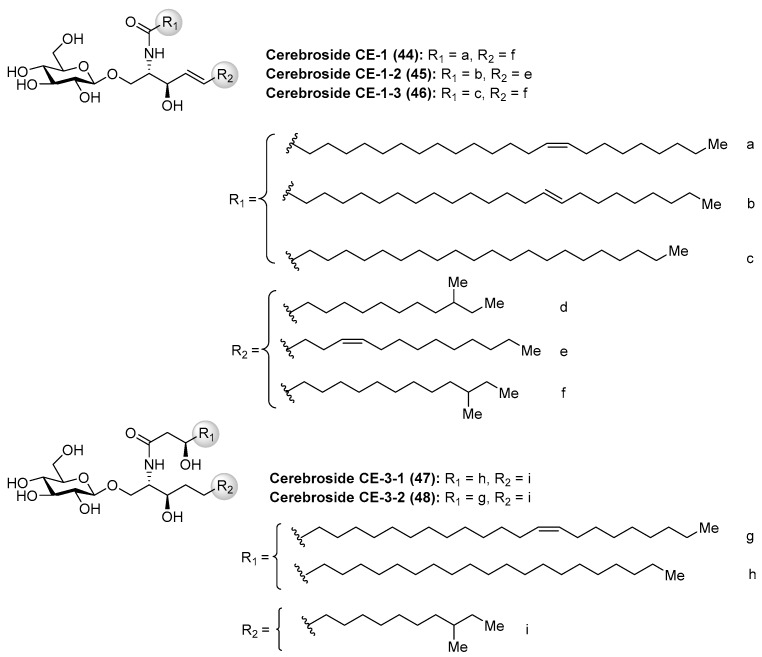
Structures of the cerebrosides CE (**44**–**48**).

Cerebrosides CE (**44**–**48**) (Figure 5) were isolated, together with ganglioside CG-1 (See Section Ganglioside CG-1), from the sea cucumber *Cucumaria echinata* [36]. These cerebrosides showed toxicity in a brine shrimp lethality assay with the rates of 11–27%. However, no further chemical and biological studies about these cerebrosides have been carried out so far.

##### Halicylindrosides

**Figure 6:**
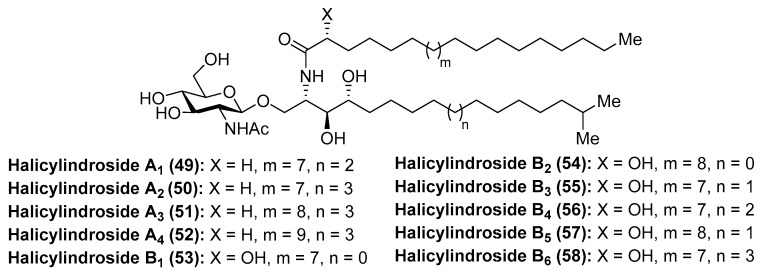
Structures of the halicylindrosides (**49**–**58**).

Within the family of cerebrosides of marine origin, the halicylindrosides (**49**–**58**) (Figure 6) were isolated and characterized by Fusetani´s group in 1995 from the marine sponge *Halichondria cylindrata* [37]. These structural studies revealed that the halicylindrosides were a new family of phytosphingosine-containing cerebrosides, which promoted a great deal of expectation by virtue of their antifungal activity against *Mortierella remanniana* at 250 µg/disk and citotoxity against P388 murine leukemia cells at 6.8 µg/mL, respectively.

**Scheme 3:**
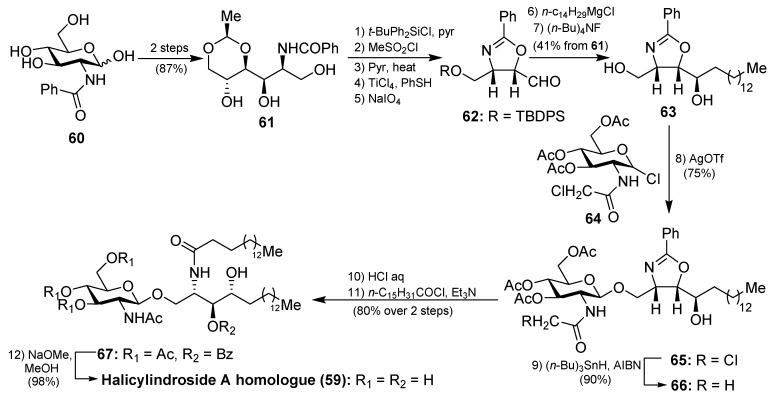
Total synthesis of halicylindroside A homologue **59**.

The synthesis of the halicylindroside A homologue **59** was carried out by Murakami et al. [38], paving the way for the synthesis of the natural congeners. Thus, **61**, which was prepared from *N*-benzoyl-d-glucosamine **60** in 87% over two steps, was transformed into the oxazoline derivative **63** in seven steps in 41% overall yield, involving regioselective *O*-methanesulfonation and diastereoselective Grignard addition as key steps. The coupling between **63** and *N*-chloroacetyl glucosyl chloride **64**, which was chosen as a more efficient donor for the glycosylation reaction, was achieved by using silver trifluoromethanesulfonate as the promoter at 70 °C to obtain **65** in 75% yield. Reduction of the chloroacetyl **65** with *n*-Bu_3_SnH afforded acetamide **66** in 90% yield. Subsequent acid-catalyzed ring opening of the oxazoline **66**, followed by reaction with palmitoyl chloride of the resulting amino alcohol provided **67**, which was finally transformed into halicylindroside A homologue (**59**) after a basic treatment (Scheme 3). In a similar manner, a homologue of halicyndroside B (**53**) was obtained by using (2*R*)-acetoxypalmitoyl imide. 

##### Phallusides 

**Figure 7:**
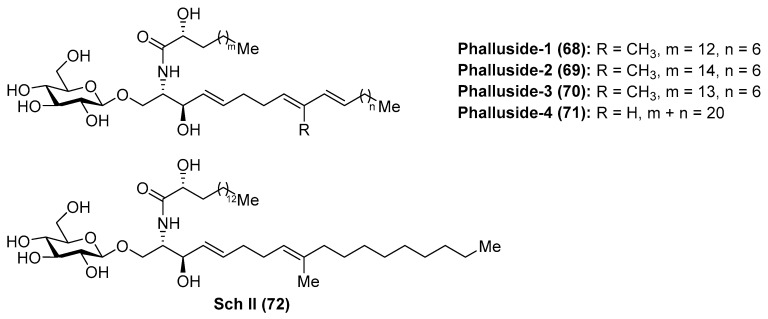
Structures of the phallusides (**68**–**71**) and Sch II (**72**).

Phallusides (**68**–**71**) (Figure 7) were isolated from the Mediterranean ascidian *Phallusia fumigata* and from the starfishes *Allostichaster inaequalis* and *Cosmasterias lurida* [39] and their structures were elucidated by a combination of spectroscopic and chemical degradation studies. These studies revealed the presence of an unusual sphingoid base, which corresponded to the 2-amino-9-methyl-d-*erythro*-(4*E*,8*E*,10*E*)-octadeca-4,8,10-triene-1,3-diol. Prior to these findings, Karlsson et al. [40] had described isolation and structure elucidation of a closely related glycosphingolipid from the sea anemone *Metridium senite*, which was assigned as Sch II (**72**). Interestingly, this compound was also isolated from the basidiomycete *Schizophyllum commune* some years later [41]. 

The biological activity evaluation of phallusides 1–3 (**68**–**70**) revealed that these compounds possessed significant activity as antifungal agents against several phytopathogenic fungi (*Fusarium oxysporum f.* sp. *Niveum*, *F. solani f.* sp. Cucurbitae, *Pythium ultimum,* and *Alternaria solani*) [42].

**Scheme 4:**
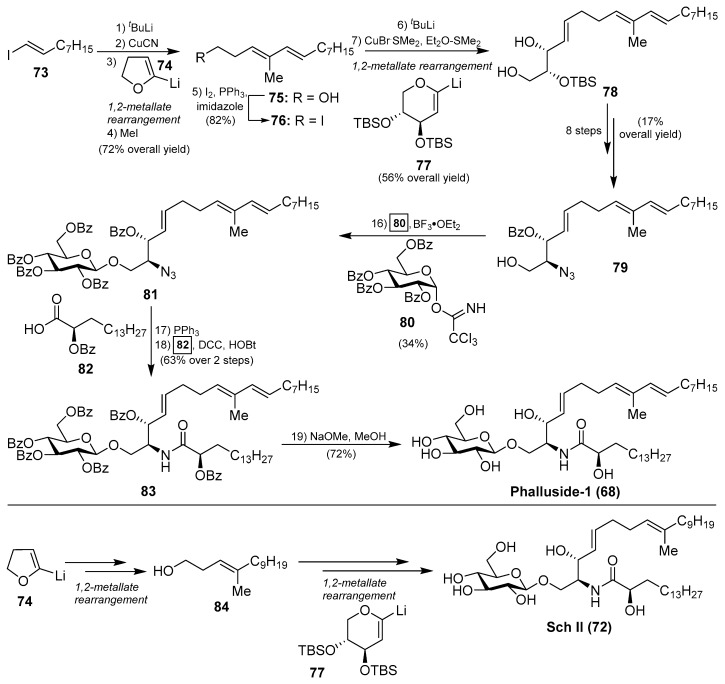
Total synthesis of phalluside-1 (**68**) and Sch II (**72**).

The first synthetic explorations of these compounds were attempted by Kocienski et al. [43] leading to the total syntheses of phalluside-1 (**68**) and Sch II (**72**) via a Cu (I)-mediated 1,2-metallate rearrangement of a lithiated glycal as a key step for the stereoselective synthesis of the sphingoid base. In this direction, the authors envisioned the synthesis of the azidosphingatrienine **79** from iodoalkene **73** through two sequentially 1,2-metallate rearrangements involving cyanocuprate intermediates, derived from lithium derivatives **74** and **77**, to obtain the triene triol **78**. Conversion of **78** into the azide **79** proceeded in eight steps in 17% overall yield. The key Schmidt glycosylation reaction of the alcohol **79** with the donor **80**, using BF_3_^.^OEt_2_ as a promoter, afforded the glycoside **81** in a modest 34% yield. To complete the synthesis, a Staudinger reaction of the azide **81**, followed by an *N*-acetylation of the resulting amine with the acid **82** generated protected phalluside **83** in 63% yield over two steps. Final debenzoylation under basic conditions gave phalluside **68** in a 72% yield. For the synthesis of Sch II (**72**), the authors employed the same synthetic strategy using the same lithium derivatives **74** and **77** but starting from a different iodoalkene (Scheme 4).

#### 2.1.2. Diosylceramides

##### Amphiceramides

**Figure 8:**
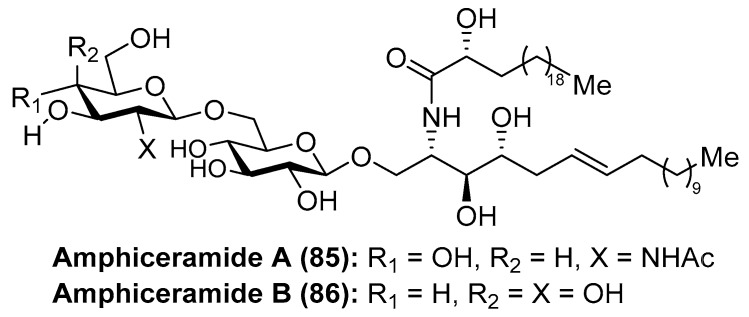
Structures of the amphiceramides A (**85**) and B (**86**).

Amphiceramides A (**85**) and B (**86**) were isolated from the Caribbean sponge *Amphimedon compressa* by Mangoni et al. [44] (Figure 8). The structures of both glycolipids contain an unusual Δ^6^-phytosphingosine unit, which can be found in other secondary metabolites such as the aforementioned axidjiferosides A–C (**41**–**43**). In addition, amphiceramide A (**85**) contains an uncommon *N*-acetyl-β-glucosamine, which has never been found in any natural product. Amphiceramide B (**86**) was the first glycosphingolipid that possesses an allolactose [Gal(1 β→6)Glc] residue β-linked to the ceramide moiety.

##### Plakosides

**Figure 9:**
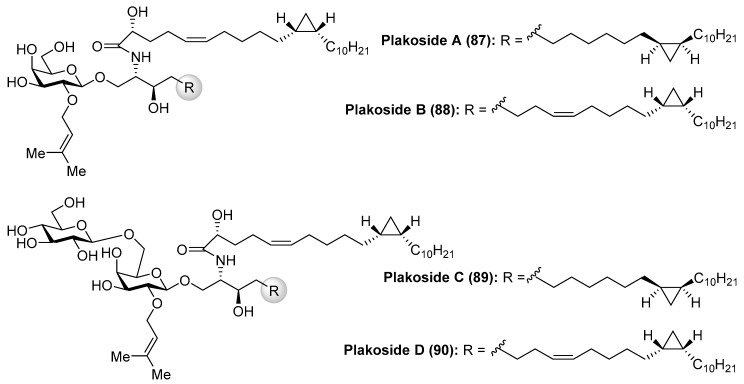
Structures of plakosides **87**–**90**.

Fattorusso et al. [45] described the isolation of plakosides (**87**–**90**) (Figure 9) from the marine sponge *Plakortis simplex,* which featured the presence of a cyclopropane ring in the lipidic chain and a 2-*O*-prenylation of the carbohydrate moiety. Interestingly, in contrast to the immunostimulating property of related glycosphingolipids, such as agelasphins, the plakosides exhibited potent immunosuppressive activity, with plakosides A (**87**) and B (**88**) as inhibitors of the proliferative response of lymph-node cells when T cells were stimulated with concavaline A in all doses tested (0.01–10 µg/mL). In addition, these natural products did not display cytotoxic property. This intriguing immunosuppressive activity was ascribed to the presence of the susbstituent on C-2 of the inner monosaccharide. Some years later, the same authors isolated two new related glycosphingolipids, plakosides C (**89**) and D (**90**), from another marine-sponge species, *Ectyoplasia ferox* [46]. Further investigations led to the conclusion that the plakosides are in fact biosynthesized by sponge-associated bacterial symbionts and not from the sponge itself [46].

**Scheme 5:**
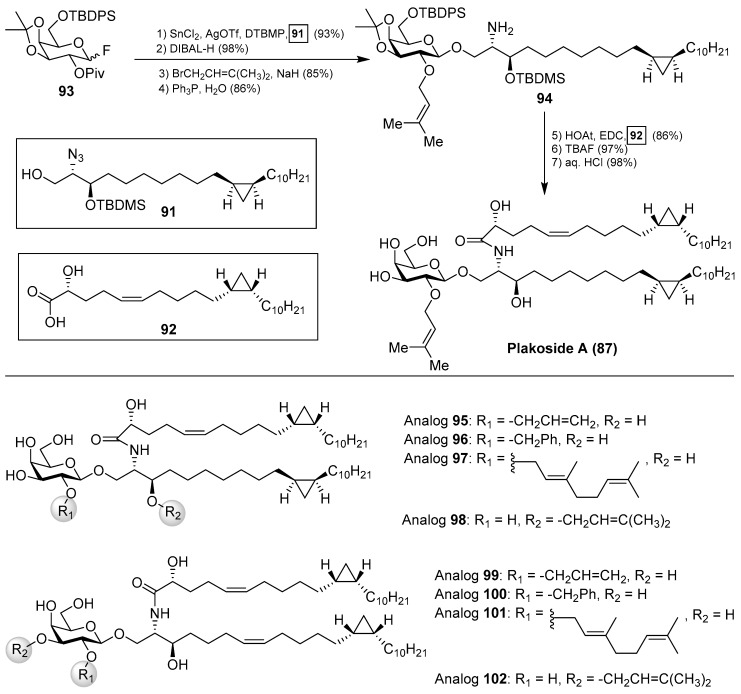
Total syntheses of plakoside A (**87**) and its analogues **95**–**102**.

The interesting molecular structures of the plakosides, combined with their intriguing immunosuppressive activity, have prompted their total synthesis to further investigate their biological properties. Thus, Nicolaou et al. [47] reported an efficient and stereoselective total synthesis of these glycosphingolipids, which provided not only sufficient amounts of the compounds for further biological studies, but also some analogues that allowed the determination of key structural factors for the immunosuppressive activity through a structure–activity-relationship study. Having prepared the cyclopropane-containing derivatives **91** and **92** in stereoselective manner, a first assembly of **91** with the corresponding galactosyl fluoride **93** was achieved in an excellent 93% yield in a stereoselective glycosylation reaction mediated by SnCl_2_/AgOTf. The introduction of the prenyl group was then carried out in two steps to obtain the key intermediate **94**, which was prepared for the final steps (Scheme 5). These steps consisted of an amide coupling between **94** and the fatty acid **92**, followed by a global protecting group deprotection in two additional steps. 

**Scheme 6:**
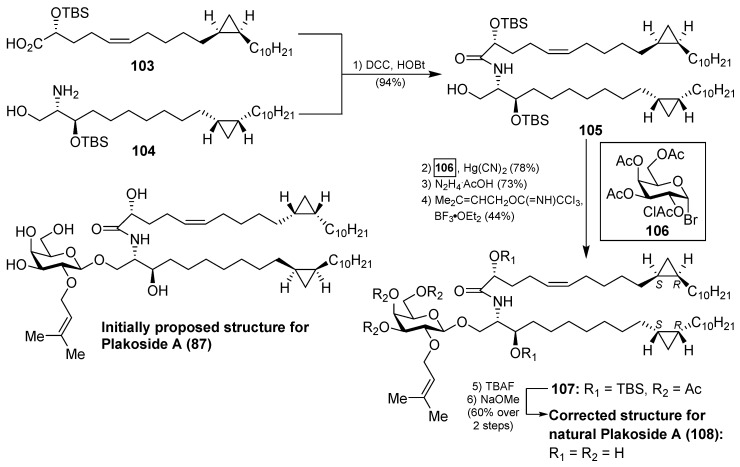
Total synthesis of the corrected structure of plakoside A (**108**).

In a similar manner, the synthesis of plakoside B (**88**) was efficiently accomplished by using the suitable sphingosine derivative. This synthetic strategy was extended to the preparation of a small collection of plakoside analogues **95**–**102**, in which the effect of the prenyl group upon the biological activities was analysed by modification of this group. In fact, with all these plakosides in hand, their biological activities were evaluated in three in vitro assays (the mixed-lymphocyte-reaction proliferation (MLR) assay, the concanavalin A response assay, and the murine bone marrow cell proliferation assay). Surprisingly, the authors found that all these compounds (**95**–**102**), and the synthetic plakosides A (**87**) and B (**88**), displayed modest immunosuppressive activity compared to the reported activities for the natural compounds. Among them, analogue **95** was the most active in the series, albeit in a modest range, with an IC_50_ value of 7.1 µM in the MLR proliferation assay. The discrepancy between the immunosuppressive activities of the synthetic plakosides and of the natural products reported in the literature could be explained by the work of Mori et al., who corrected the absolute configuration initially assigned for the plakosides [48,49]. These authors demonstrated, through their total synthesis described in Scheme 6, that the absolute configurations of the stereogenic carbons of the cyclopropane rings present in both lipidic chains, through compounds **103** and **104**, were opposite to those initially proposed by the Fattorusso’s group [50,51]. The incorrect configurations assigned for plakoside A (**108**) could explain the poor immunosuppressive activity exhibited by the synthetic plakosides A and B prepared by Nicolaou et al. which corresponded to the stereoisomers of the naturally ocurring counterparts. It is interesting to point out that the structures of the synthetic plakosides obtained by Nicolaou’s group possessed the same spectroscopic data and optical rotations as those from naturally occurring plakosides. This fact led them to conclude that the synthetic plakosides were the same as the naturally occurring counterparts.

##### Terpiosides

**Figure 10:**
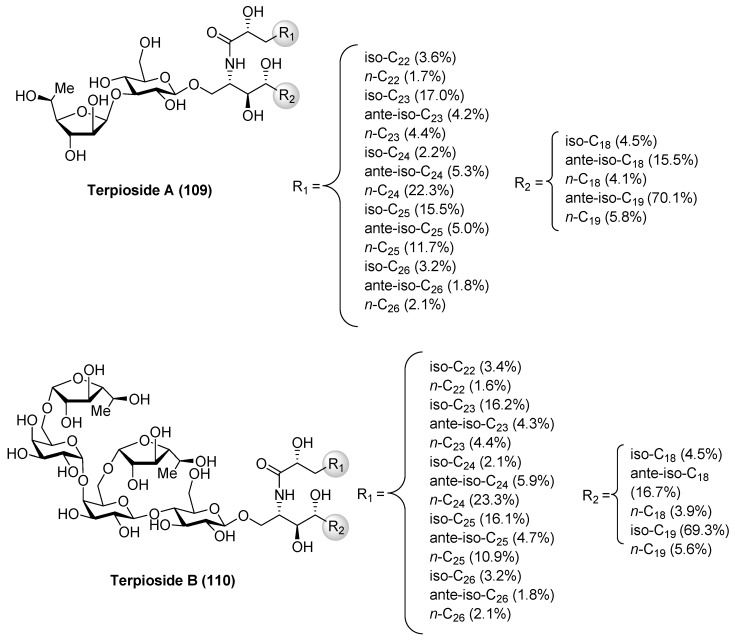
Structures of terpiosides A (**109**) and B (**110**).

Terpioside A (**109**) and B (**110**) (Figure 10) were isolated from the marine sponge *Terpios* sp. by Costantino et al. [52] and represent the first glycosphingolipids reported from sponges of the genus *Terpios*. Later, Cutignano et al. [53] reported the isolation of the terpiosides from the Antarctic sponge *Lyssodendoryx flabellata*. The structure of terpioside A (**109**) was secured by a combination of extensive spectroscopic analysis, as well as chemical degradation studies, revealing the presence of a unique sugar moiety, comprised of an α-fucofuranoside, linked to the 3-position of a β-glucopyranoside, which is the sugar residue linked to the ceramide. Thus, terpioside A (**109**) represents the first natural glycosphingolipid that contains an l-fucose in a furanose form. On the other hand, terpioside B (**110**) contains a pentasaccharide chain, which possesses two terminal α-l-fucofuranose units. The biological activity evaluation of terpioside B (**110**) led to the discovery that this compound was capable of inhibiting LPS-induced NO release, displaying higher activity than simpler glycosphingolipids, such as terpioside A (**109**) and monoglucosylceramide [54].

#### 2.1.3. Neutral Glycosphingolipids with Oligosaccharide Chains

##### Agelagalastatin

Agelagalastatin (**111**) was isolated from the Western-Pacific marine sponge *Agelas* sp. as a mixture of two isomers (**111a** and **111b**) through a human cancer cell line bioassay carried out by Pettit et al. [55]. Agelagalastatin showed significant in vitro antitumoral activity against various human cancer cell lines, including lung NCI-H460, brain SF-295, renal A498, colon KM20L2, melanoma SK-MEL-5, and the ovarian OVCAR-3, with a range of GI_50_ values from 0.77 µg/mL to 2.8 µg/mL. Structurally, this natural product contains an unprecedented digalactofuranosyl unit, which has not been found before in a natural product.

**Scheme 7:**
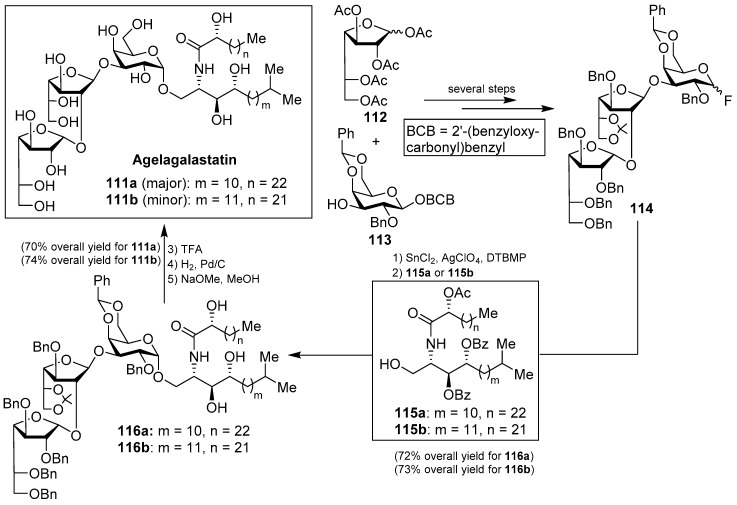
Structures of agelagalastatins (**111a** and **111b**) and their total synthesis.

This intriguing structure, in conjuction with its antitumor activity, drew the attention of many synthetic chemists and, as a consequence, a total synthesis of agelagalastatin (**111**) was reported by Kim et al. [56] based on an α-selective glycosylation of the ceramide **115a** or **115b** with the trisaccharide fluoride **114**, prepared from monosaccharide derivatives **112** and **113** after several steps. This key glycosylation reaction, performed in the presence of SnCl_2_, AgClO_4_, and DTBMP, furnished **116a** and **116b** in 72 and 73% yields, respectively. Completion of the synthesis involved the final deprotection of the benzyl, *O*-acetyl, and *O*-benzoyl groups in 70 and 74% overall yields to obtain agelagalastatins **111a** and **111b**, respectively (Scheme 7).

##### Clarhammosides

**Figure 11:**
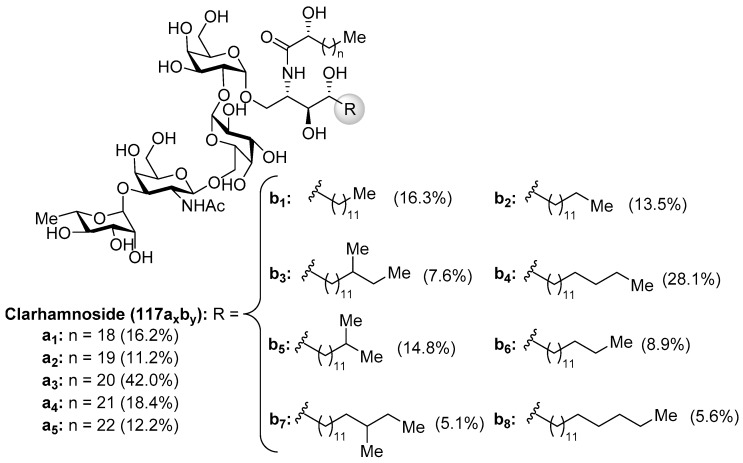
Structures of the clarhamnosides (**117a_x_b_y_**).

The clarhamnosides **117a_x_b_y_** (Figure 11) were isolated as an inseparable mixture of different members from the marine sponge *Agelas clathrodes* by Costantino et al. [57]. The combination of 2D NMR and CD spectroscopic techniques allowed the structural determination of this complex glycosphingolipid mixture. Clarhamnosides are the first α-galactoglycosphingolipids with a l-rhamnose unit in the sugar head. In addition, the sequential two 1,2-*cis*-α-d-galactopyranosidic linkages are also rare in nature.

**Scheme 8:**
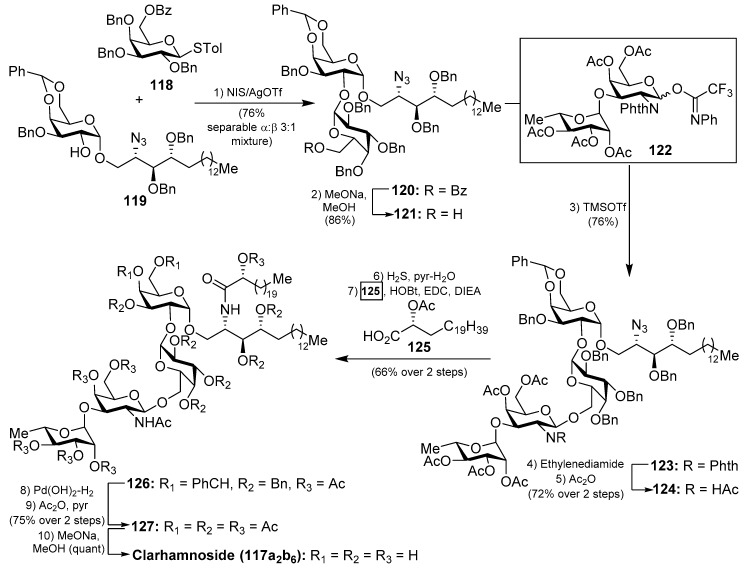
Total synthesis of clarhamnoside **117a_2_b_6_**.

The first total synthesis of a clarhamnoside member (**117a_2_b_6_**) was carried out by Li et al. [58]. According to this work, the glycosylation reaction between **118** and **119**, promoted by the NIS/AgOTf system, afforded a 3:1 α:β separable mixture of the disaccharide **120** in 76% yield. Removal of the benzoyl group in **120** gave the disaccharide **121** in 86% yield, which was used as an acceptor for the next glycosylation reaction. In this reaction, **121** was reacted with donor trifluoroacetimidate **122** employing TMSOTf and 4 Å MS at −20 °C to provide the β-glycoside **123** in 76% yield. Conversion of the *N*-phthalimido group into an acetamido group by sequential treatment with ethylenediamine and Ac_2_O afforded **124** in 72% yield in two steps. The reduction of **124**, employing H_2_S in pyridine/water, was prior to the corresponding coupling of the resulting amine with α-hydroxy acid **125** to provide **126** in 66% yield in two steps. Final hydrogenolysis afforded the corresponding tetrasaccharide intermediate, which was acetylated to obtain clarhamnoside peracetate **127**. Removal of the acetyl groups furnished clarhamnoside **117a_2_b_6_** in a moderately good yield (Scheme 8).

##### Vespariosides

**Figure 12:**
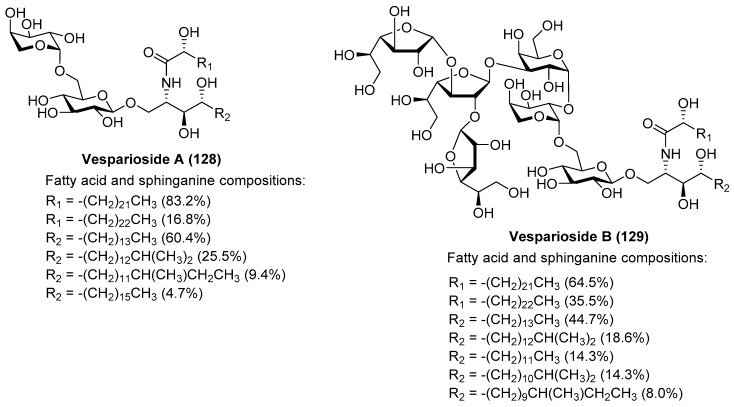
Structures of vespariosides A (**128**) and B (**129**).

After the isolation of vesparioside A (**128**) by Mangoni et al. [59] from *Spheciospongia vesparia* in 2005, the same authors in 2008 reported the isolation and structural elucidation of a unique furanose-rich glycosphingolipid that was named vesparioside B (**129**) (Figure 12) [60]. Through an exhaustive spectroscopic analysis, supported by theoretical and degradation studies, the authors were able to establish the absolute configuration of this stunning molecule, which was unambiguously confirmed through the total synthesis carried out by Yang et al. in 2016 [61]. In this total synthesis, after an extensive exploration of various strategies, the authors decided to construct initially the glycosphingolipid derivative **133**, via a glycosylation reaction of the donor trichloroacetimidate **131**, prepared from the disaccharide **130**, and the phytosphingosine acceptor **132**. 

**Scheme 9:**
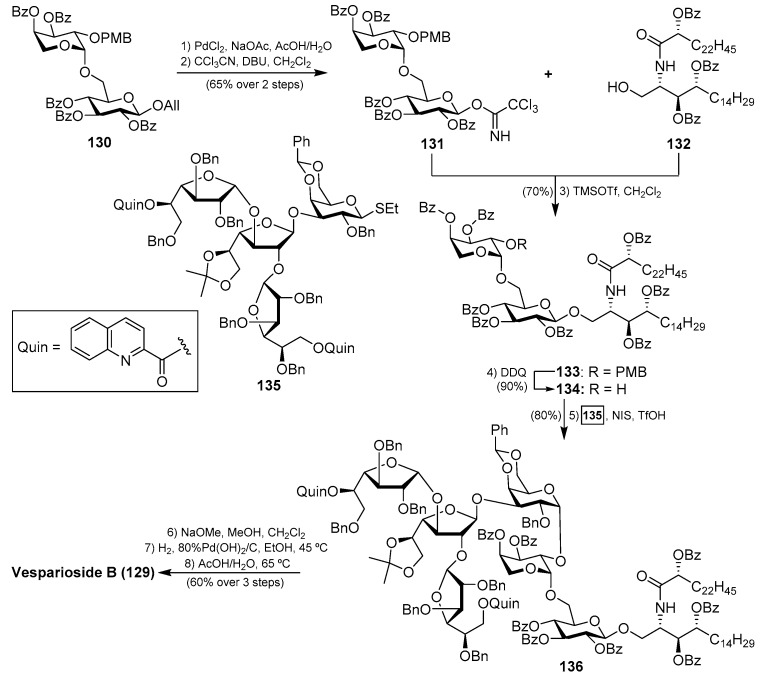
Total synthesis of vesparioside B (**129**).

The resulting glycosphingolipid **133** was then prepared for assembly with the tetrasaccharide fragment in the form of thioglycoside **135**. To this end, the removal of the PMB group of **133** by treatment with DDQ provided the alcohol **134**, which was reacted with the donor **135** under the action of the promoter NIS/TfOH. The resulting complex glycosphingolipid **136**, obtained in excellent yield and stereoselectively, was then transformed into natural vesparioside B (**129**) after removal of all the protecting groups in a 60% overall yield in three steps (Scheme 9). 

**Scheme 10:**
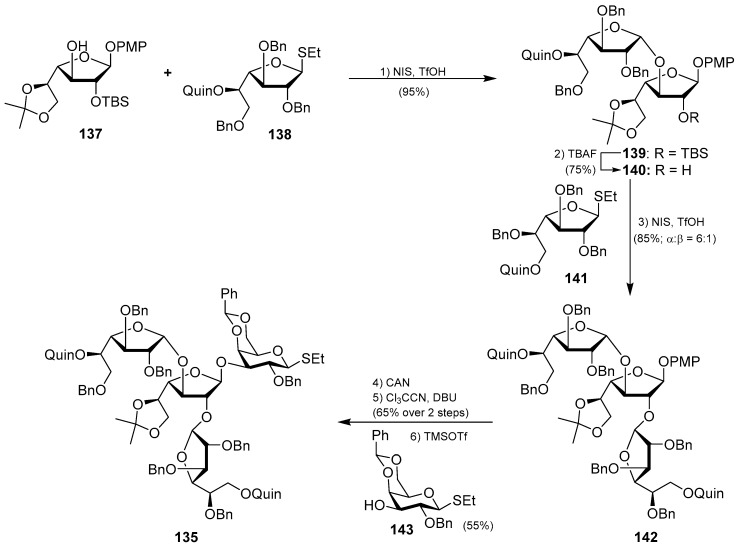
Synthesis of the tetrasaccharide unit **135** of vespariosides.

Interestingly, the preparation of the tetrasaccharide **135** required the stereoselective synthesis of 1,2-*cis*-α-galactofuranoside units, for which some methods have been recently developed. In this case, Yang’s group has developed an elegant methodology based on a hydrogen-bond-mediated aglycone delivery (HAD) strategy for which they employed a 2-quinolinecarbonyl (Quin) functionality. This group, acting as an H-bond acceptor and incorporated in the α-face of the galactofuranoside ring, displays a strong α-stereocontrol effect in glycosylation reactions when furanosyl thioglycoside donors are employed in reactions with various acceptors. The application of this strategy in the synthesis of tetrasaccharide **135** commenced with the glycosylation reaction of the readily available galactofuranosyl acceptor **137** and the donor **138**, which was armed with the Quin group at the 5-position. This reaction delivered exclusively the α-disaccharide **139** in an excellent 95% yield, proving the reliability and validity of this strategy. The assembly of the third galactofuranosyl derivative proved more challenging, as the use of the 5-*O*-Quin derivative **138** as a donor did not work, resulting in recovered starting materials. Fortunately, when the 6-*O*-Quin derivative **141** was used instead, the reaction afforded the expected trisaccharide **142** as a 6:1 α/β separable mixture in an 85% combined yield. Finally, the fourth glycoside was incorporated through a glycosylation reaction with the trichloroacetamidate derivative of **142** and the thioglycoside acceptor **143** to deliver tetrasaccharide **135** in a 55% yield as a single β-anomer (Scheme 10). This total synthesis not only confirmed the molecular structure of this fascinating glycosphingolipid, but also, more importantly, provided the opportunity for further biological exploration of this class of compounds, which was not achievable due to the scarcity of material from natural sources. These biological studies with synthetic vespariosides are currently in progress.

### 2.2. Acidic Glycosphingolipids

#### 2.2.1. Gangliosides

Structurally characterized by the presence of one or more sialic acid units, the gangliosides of a marine origin are almost exclusively found in echinoderms, mainly from urchins. In other marine invertebrates, it has been demonstrated that the sialic acid unit is replaced by a glucuronic acid residue. Despite the limited distribution of this class of glycosphingolipids in the marine realm, a huge number of natural products belonging to this category has been discovered. As a consequence, we have selected for this section the most biologically relevant gangliosides.

##### Ganglioside Hp-s1

**Figure 13:**
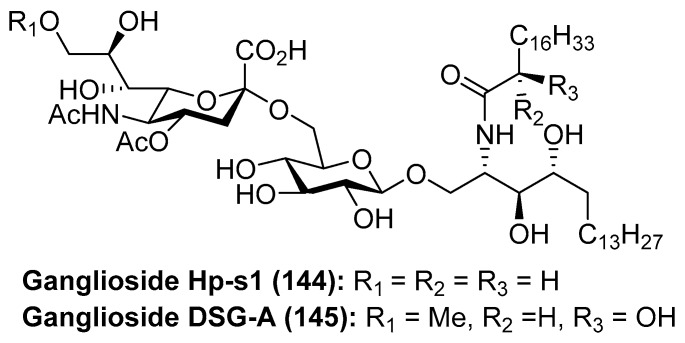
Structures of ganglioside Hp-s1 (**144**) and DSG-A (**145**).

Ganglioside Hp-s1 (**144**) was isolated from the ovary of the sea urchin *Diadema setosum* or from the sperm of the sea urchin *Hemicentrotus pulcherrimus* [62]. This compound showed an interesting neuritogenic activity towards the rat pheochromocytoma PC-12 cell line in the presence of nerve growth factor (NGF) and, when compared to ganglioside GM-1 (25.4%), its effect was better (34.0%). Together with ganglioside Hp-s1, ganglioside DSG-A (**145**) (Figure 13), isolated also from *D. setosum*, displayed similar neuritogenic activity, which can be useful for the treatment of neurodegenerative diseases [63].

**Scheme 11:**
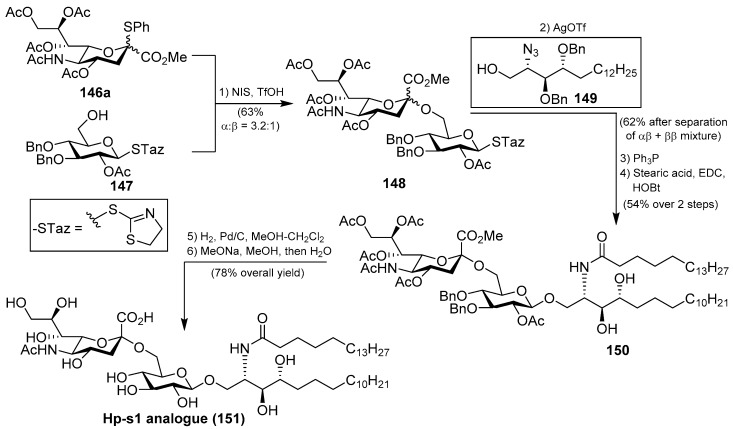
Total synthesis of Hp-s1 analogue **151**.

Several syntheses of these natural products have been reported [64]. Initial synthetic efforts were carried out by Tsai et al. [65], who prepared the ganglioside Hp-s1 analogue **151**. The synthesis was accomplished by a sequence of chemoselective glycosylation reactions as key steps. Thus, the disaccharide **148** was obtained by the sialylation of the acceptor **147** with the sialyl donor **146a**, mediated by NIS/TfOH as a promoter at −30 °C in the presence of 4 Å molecular sieves, in 63% yield as an inseparable α:β 3.2:1 mixture. The next glycosylation reaction involved the resulting disaccharide **148** with the azidosphingosine derivative **149** in the presence of AgOTf and 3 Å molecular sieves at room temperature to obtain the resulting disaccharide derivative as a separable mixture of αβ and ββ anomers in 62% combined yield. From the corresponding αβ anomer, a Staudinger reaction was followed by an amidation process to obtain **150** in 54% yield in two steps. Final debenzylation and deacetylation reactions afforded the ganglioside Hp-s1 analogue **151** in 78% overall yield (Scheme 11). This analogue exhibited neurogenetic activity towards the human neuroblastoma cell line SH-SY5Y without the presence of NGF.

**Scheme 12:**
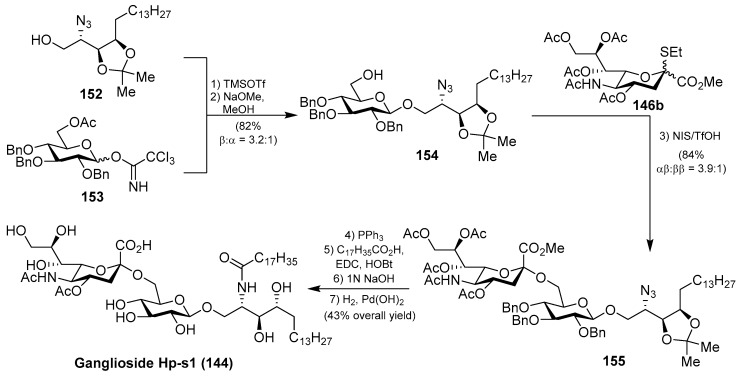
Total synthesis of ganglioside Hp-s1 (**144**).

A different synthetic strategy was used in the first total synthesis of ganglioside Hp-s1 (**144**), achieved by Luo et al. [64], based on two key glycosylation reactions. According to this synthetic strategy, after an extensive optimization of the glycosylation reactions, the authors found that the first reaction of phytosphingosine **152** with benzyl protected imidate **153**, using TMSOTf as a promoter at −30 °C to room temperature, followed by a deacetylation step, afforded **154** in 82% (β:α 3.2:1) yield. The second glycosylation reaction involved the glycosyl acceptor **154** and the sialyl donor **146b**, mediated by NIS/TfOH as a promoter, to obtain **155** in 84% yield (αβ:ββ 3.9:1). From **155**, the conversion of the azide group into the corresponding amine, through a Staudinger reaction, followed by amide formation, and final deprotection of acetyl, benzyl, and acetonide groups delivered ganglioside Hp-s1 (**144**) in 43% overall yield from **155** (Scheme 12).

**Figure 14:**
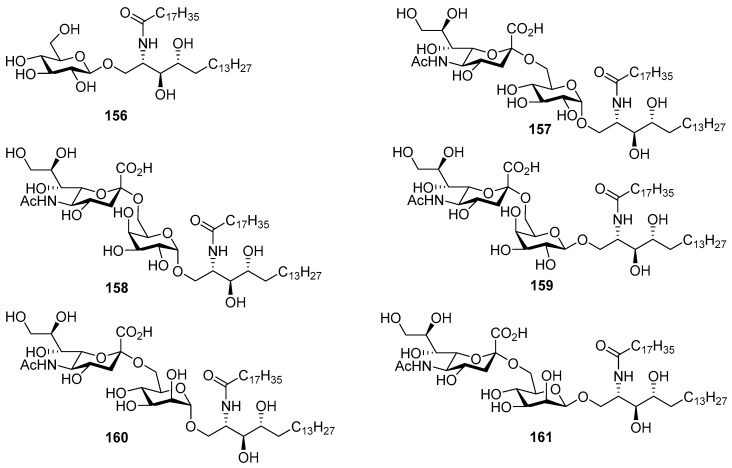
Gangliosides Hp-s1 analogues **156**–**161**.

The same authors carried out an SAR study involving the synthesis of six Hp-s1 analogues (**156**–**161**) by replacing the glucosyl unit of Hp-s1 with α-glucose (**157**), α- and β-galactose (**158** and **159**), and α- and β-mannose (**160** and **161**), including the simple cerebroside **156** [66]. After the biological evaluation, the authors found that the C-2 hydroxyl group of the glucosyl unit played a crucial role in the stimulation of neurite outgrowth of SH-SY5Y cells. In addition, it was found that analogue **158** activated NKT cells, although it was inactive on neurite outgrowth of SH-SY5Y cells (Figure 14).

Based on these synthetic studies, the same authors developed an efficient method for α-selective sialylation based on a pre-activated 5-*N*,4-*O*-carbamate thiosialoside donor using *p*-TolSCl/AgOTf as promoters which was extended to the synthesis of gangliosides Hp-s1 and DSG-A [67]. Tsai et al. also achieved the total synthesis of ganglioside DSG-A (**145**) through a chemoselective glycosylation reaction in a [1 +1 + 2] synthetic strategy for the assembly of the four fragments of this molecule [68]. 

##### Gangliosides HLG

**Figure 15:**
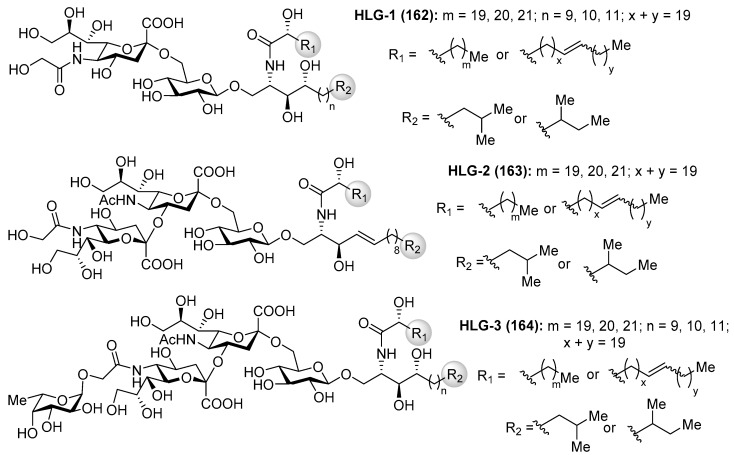
Structures of gangliosides HLG-1, -2, and -3 (**162**–**164**).

Gangliosides HLG-1 (**162**), HLG-2 (**163**) and HLG-3 (**164**) (Figure 15) were isolated from the sea cucumber *Holothuria leucospilota* by Higuchi et al. [69]. These compounds showed similar neuritogenic activity toward the rat pheochromocytoma cell line, PC-12 cell, in the presence of NGF as other marine-derived gangliosides. Structurally, these gangliosides contain a unique α(2,4) linkage between sialic acids, which captured the attention of many synthetic chemists.

**Scheme 13:**
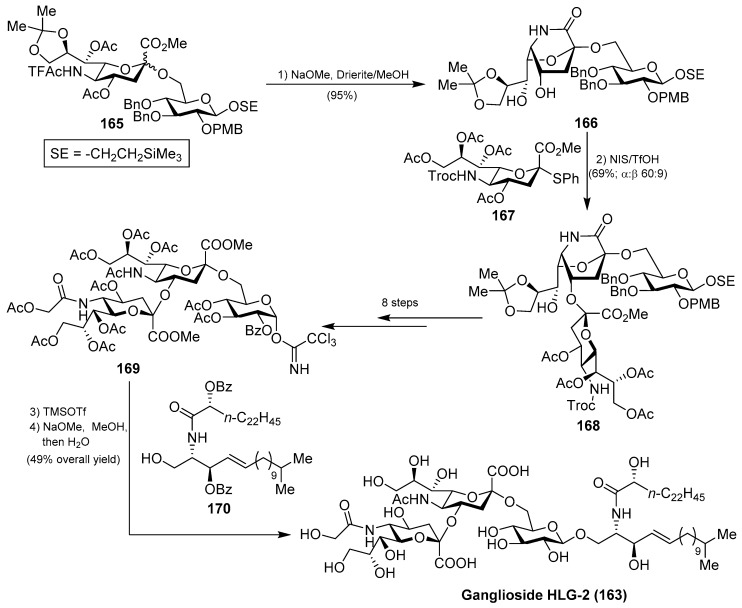
Total synthesis of ganglioside HLG-2 (**163**).

The first total synthesis of ganglioside HLG-2 (**163**) was carried out by Kiso et al. [70]. To construct the glycan unit, sialylation at C-4 hydroxyl group of the sialic acid residue was achieved using the 1,5-lactam-sialyl unit **166** as a novel glycosyl acceptor and readily prepared from **165** by a basic treatment (refluxing NaOMe in MeOH) in 95% yield. The glycosylation reaction of **166** with Troc-sialyl donor **167** in the presence of NIS/TfOH took place exclusively at C-4 hydroxyl group in a stereoselective manner to give the trisaccharide **168** in 69% yield (α:β 60:9). To complete the synthesis, the next glycosylation reaction involved the donor **169**, obtained in eight steps from the trisaccharide **168**, and the ceramide acceptor **170** in the presence of TMSOTf as a promoter, to obtain the corresponding ganglioside in 49% yield, which was finally transformed into ganglioside HLG-2 (**163**) in a quantitative yield after a basic treatment (Scheme 13).

**Scheme 14:**
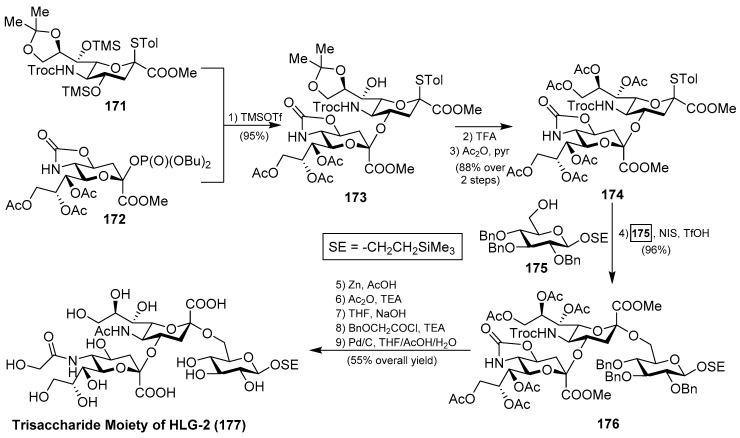
Total synthesis of the trisaccharide core **177** of ganglioside HLG-2 (**163**).

Interesting was the synthetic approach by Ye et al., who achieved the synthesis of the glycan portion of ganglioside HLG-2 in a highly efficient and stereoselective manner, involving two key glycosylation reactions: first, for the construction of the disaccharide **173** using the phosphate donor **172**, in the presence of TMSOTf, in 95% yield; and, second, for the generation of the trisaccharide **176** by the use of NIS/TfOH in 96% yield, both with excellent α-stereoselectivity. From **176**, final conversion of functional groups and deprotection reactions afforded the trisaccharide core of ganglioside HLG-2 (**177**) [71] (Scheme 14).

##### Ganglioside GP-3

Ganglioside GP-3 (**178**) was isolated from the starfish *Asterina pectinifera* by Higuchi et al. [72]. As previous gangliosides, GP-3 also exhibited neuritogenic activity toward the rat pheochromocytoma PC-12 cells, in the presence of the NGF, displaying, in this case, a lower effect when compared with ganglioside GM-1. Structurally, the glycan part contains nine monosaccharide units in a unique saccharide sequence with two internal sialic acid residues and three furanose residues. 

**Scheme 15:**
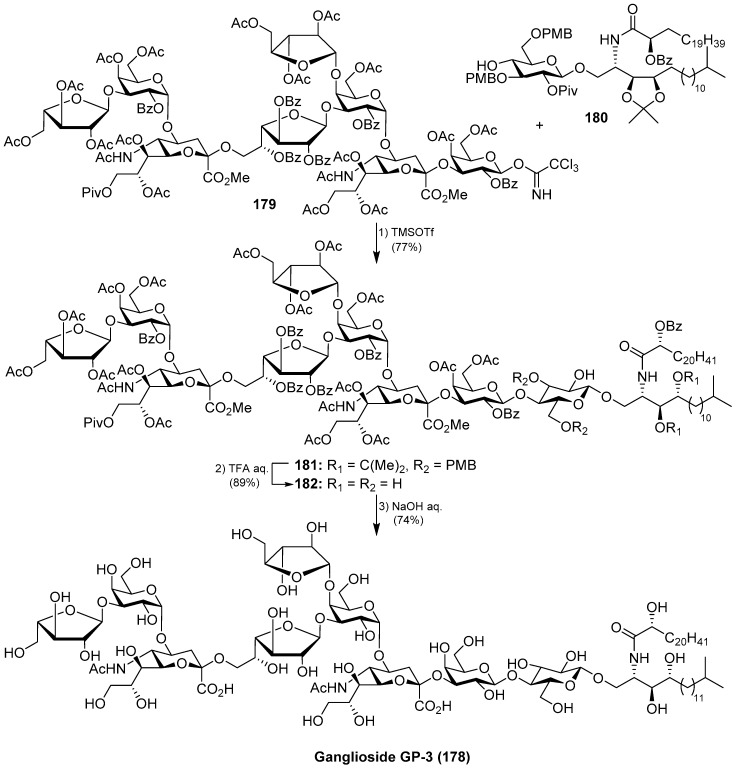
Total synthesis of ganglioside GP-3 (**178**).

The total synthesis of ganglioside GP-3 (**178**) was achieved by Kiso et al. [73]. Briefly, the final glycosylation reaction of the octasaccharide donor **179** with the glucosphingolipic derivative **180** was promoted by TMSOTf to furnish the protected ganglioside GP-3 **181** in 77% yield. A final deprotection sequence, which included aqueous TFA and basic treatments, afforded ganglioside GP-3 (**178**) (Scheme 15).

##### Ganglioside CG-1

**Scheme 16:**
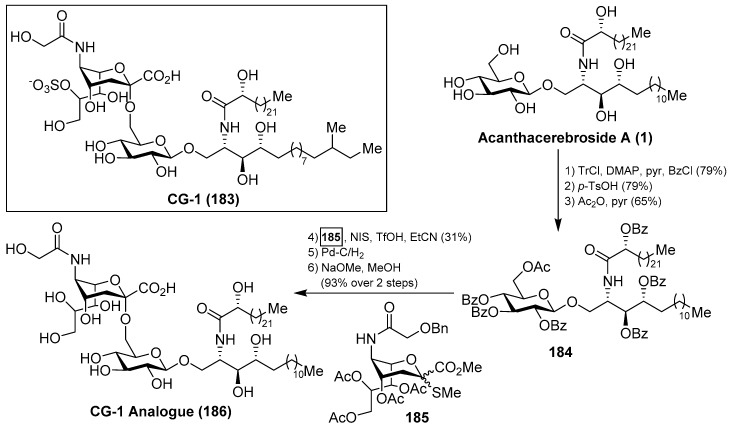
Total synthesis of ganglioside CG-1 analogue **186**.

Ganglioside CG-1 (**183**), isolated from the sea cucumber *Cucumaria echinata* by Higuchi et al. [36], showed neuritogenic activity toward the rat pheochromocytoma PC-12 cells. The same authors carried out a partial synthesis of a related CG-1 ganglioside, which lacked the sulfate group in the natural product, by a coupling of 2-thioglycoside **185** with **184**, which was prepared from the natural acanthacerebroside (**1**), in the presence of NIS/TfOH, to obtain the corresponding α-sialoside in 31% yield. This advanced precursor of CG-1 was then subjected to stepwise deprotections to obtain the related CG-1 ganglioside **186** in 93% yield [74] (Scheme 16). This synthetic study can delineate the path towards the total synthesis of this natural product.

##### Gangliosides SJG

**Figure 16:**
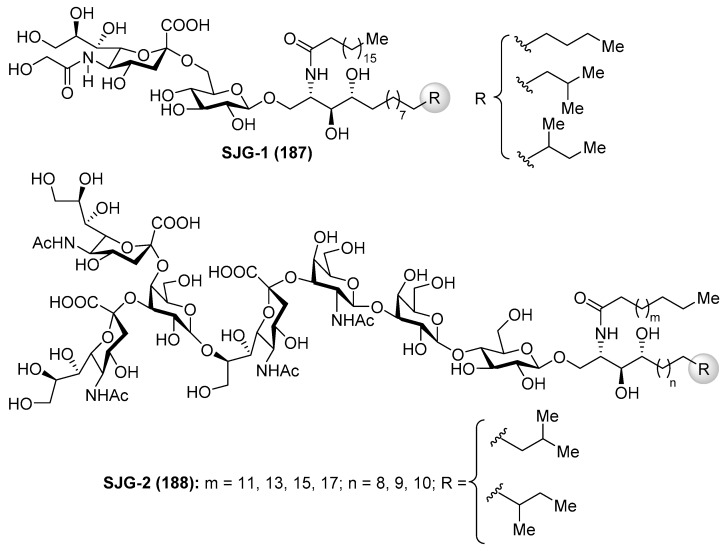
Structures of gangliosides SJG (**187** and **188**).

Gangliosides SJG-1 (**187**) and SJG-2 (**188**) were isolated from the sea cucumber *Stichopus japonicus* by Higuchi et al. [75,76] (Figure 16). As the gangliosides described in previous sections, the SJG gangliosides also showed neuritogenic activity toward the rat pheochromocytoma PC12 cells in the presence of NGF. The effect of SJG-2 was higher than that of mammalian ganglioside (proportion of neurite bearing cells SJG-2 64.8%, SJG-1 35.4%, and GM-1 47.0%).

The same authors carried out an SAR study of a few gangliosides from Echinoderms involving trisialo-gangliosides (SJG-2 and LLG-5), disialo-gangliosides (LLG-3 [77], GAA-7 [78], HLG-2, LMG-4, HLG-3, and GP-3), and monosialo-gangliosides (AG-2, AG-3, HLG-1, SJG-1, LMG-2). Evaluation of the neuritogenic activity on a rat pheochromocytoma cell line (PC-12 cells) indicated that (a) the presence of sialic acid is essential; (b) the presence of two terminal sialic acids is key for strong activity; (c) gangliosides having an 8-*O*-Me sialic acid showed stronger activity; (d) gangliosides possessing sialic acid inside of the oligosaccharide unit showed better activity; (e) the different activity showed by HLG-1 and SJG-1 (44.7 and 35.4%, respectively), which have the same sugar unit, is due to the difference in the structure of their ceramide units; (f) SJG-2, LLG-5, LLG-3, and GAA-7 have better effect than mammalian ganglioside GM-1, which has positive effects in neuritogenical diseases; and (g) these gangliosides showed no activity without the presence of NGF [79]. 

##### Gangliosides CEG

**Figure 17:**
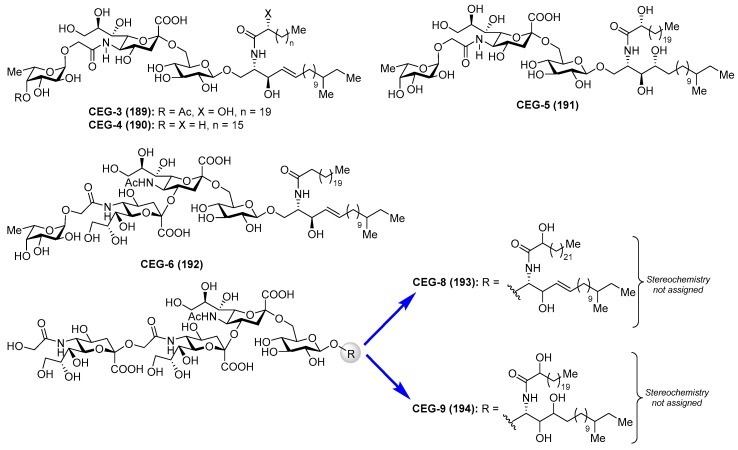
Structures of gangliosides CEG (**189**–**194**).

Gangliosides CEG-3 (**189**), CEG-4 (**190**), CEG-5 (**191**), CEG-6 (**192**), CEG-8 (**193**), and CEG-9 (**194**) were isolated from the sea cucumber *Cucumaria echinata* by Higuchi et al. [80,81] (Figure 17). These compounds showed neuritogenic activity toward the rat pheochromocytoma cell line PC-12 in the presence of a nerve growth factor.

#### 2.2.2. Sulfatides

##### Axinelloside A

**Figure 18:**
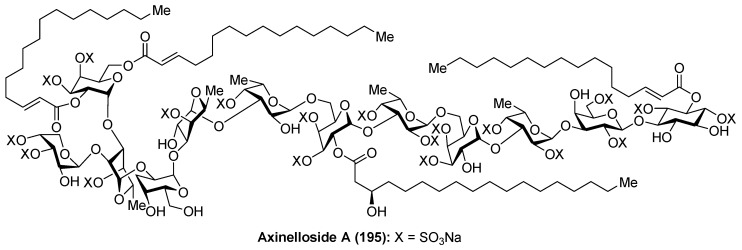
Structure of axinelloside A (**195**).

Axinelloside A (**195**), a highly sulfated liposaccharide, was isolated from the lipophilic extract of the Japanease marine sponge *Axinella infundibula* by Fusetani et al. [82]. The structure of axinelloside A (**195**) was elucidated after an excellent spectroscopic work based on 2D NMR and MS techniques, determining the presence of twelve sugars including *scyllo*-inositol, d-arabinose, five d-galactoses, and five l-fucose units, to which one (*R*)-3-hydroxy-octadecanoic acid, three molecules of (*E*)-2-hexadecenoic acids and 19 sulfates groups were attached. This compound shows similar structure to sulfated polysaccharides from the marine sponge *Chondrilla nucula* and *Dysidea fragilis* (Figure 18). 

From a biological standpoint, axinelloside A (**195**) strongly inhibited the activity of human telomerase with an IC_50_ value of 0.4 µM (2 µg/mL). In this biological activity, it is possible that the sulfate groups play a key role, since it was proven that the dictyodendrins [83], a family of sulfated pyrrolocarbazoles isolated from the sponge *Dictyodendrilla verongiformis*, lost all telomerase inhibitory activity when their sulfate groups were removed. The mechanism of action of the telomerase inhibitory activity of axinelloside A (**195**) remains unknown.

Encouraged by the telomerase inhibitory activity of axinelloside A (**195**), Walczak et al. [84] achieved the synthesis of the *scyllo*-inositol fragment from d-glucose through a Ferrier rearrangement of a vinyl-acetate derivative and stereoslective reduction of the resulting ketone. The same authors established a synthetic sequence for the preparation of a series of simple sulfated d-galactosyl liposaccharides, inspired by axinelloside A (**195**), in order to evaluate their potential as new telomerase inhibitors [85]. However, the biological activities of these galactose derivatives have not been reported so far.

## 3. Chemistry and Biology of Glycoglycerolipids

### 3.1. Aminoglycoglycerolipids and Related Glycoglycerolipids

**Scheme 17:**
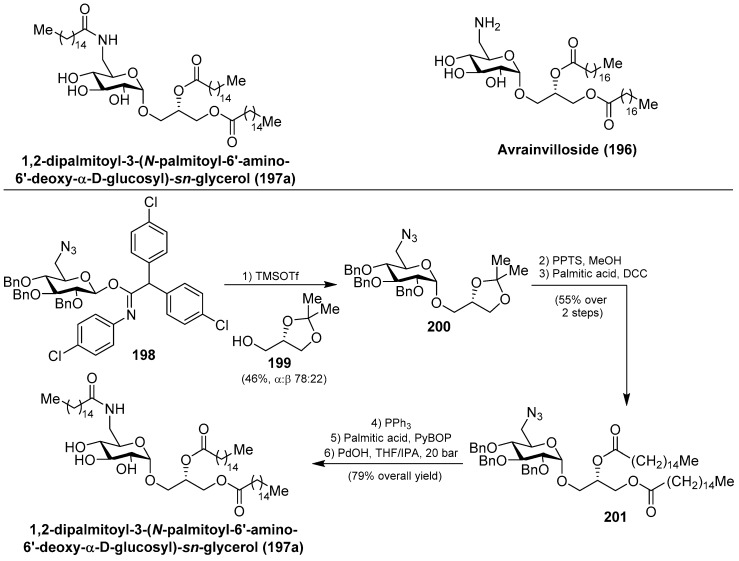
Synthesis of 1,2-dipalmitoyl-3-(*N*-palmitoyl-6′-amino-6′-deoxy-α-d-glucosyl)-*sn*-glycerol **197a**.

Within this class of glycoglycerolipids, it is noteworthy to mention avrainvilloside (**196**), isolated from the Dominican green alga *Avrainvillea nigricans* by Taglialatela-Scafati et al. [86]. Related to avrainvilloside (**196**), the aminoglycoglycerolipid 1,2-dipalmitoyl-3-(*N*-palmitoyl-6′-amino-6′-deoxy-α-d-glucosyl)-*sn*-glycerol (**197a**) was isolated from a marine alga, designated as UM2972M, by Kingston et al. This compound displayed potent inhibitory activity against the Myt1-kinase (IC_50_ = 0.12 mg/mL), an enzyme involved in the regulation of the cdc2/cyclin B kinase activity, which is essential for the growth of tumor cells [87]. Schmidt et al. [88] achieved the total synthesis of aminoglycoglycerolipid **197a** by glycosylation reaction of the imidate **198** and the alcohol **199**, promoted by TMSOTf, to obtain **200** in a 46% combined yield, as a separable α:β 78:22 mixture of anomers. Deprotection of the major α-anomer **200**, followed by an acylation step to introduce a palmitic-acid unit, afforded **201** in 55% yield in two steps. The final Staudinger reaction, followed by an amide coupling with the last palmitic acid unit and global deprotection, gave aminoglycoglycerolipid **197a** in 79% overall yield (Scheme 17).

**Figure 19:**
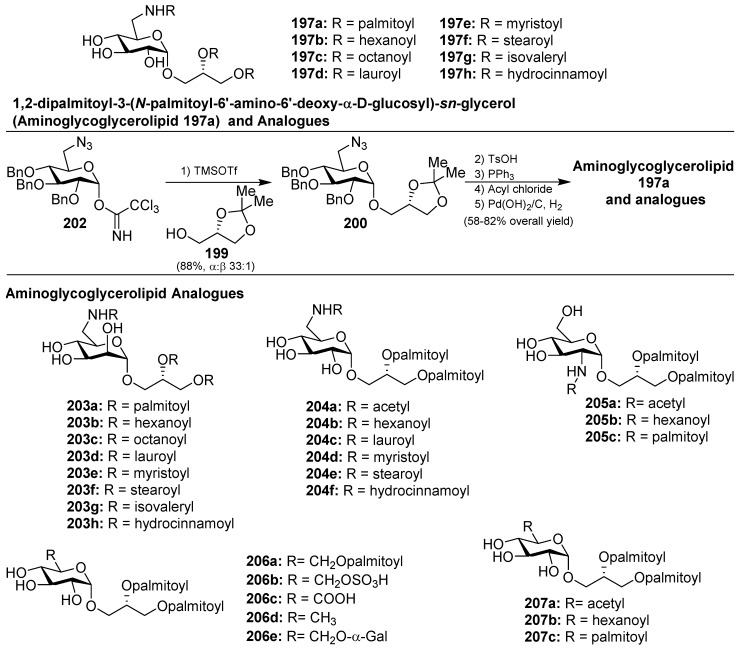
Structures of aminoglycoglycerolipids based on **197a**.

Later, Li et al. [89] completed the synthesis of the aminoglycoglyceropilpid **197a** as well as its acyl analogues (**197b**–**h**) in an efficient method employing the trichloroacetimidate donor **202** to obtain **200**, by reaction with the glycerol derivative **199**, in 88% yield and high α-selectivity (α:β 33:1) as the key step. The same authors synthesized different mannosyl (**203a**–**h**) and galactosyl analogues related to **197** in a similar manner. Given the antiviral activity displayed by many glycoglycerolipids isolated from algae, the authors evaluated the inhibitory activity of these amino derivatives against the influenza A virus (IAV) by the cytophatic effects inhibition assay. These analogues displayed inhibition of the viral replication in MDCK cells and it was found that the type of the linkages and the length of the acids could influence the activity. Among them, **203g** showed the best activity with an IC_50_ of 69.9 µM [90,91]. Later, new analogues **204**–**207** (Figure 19) were prepared and tested for anti-IAV activity, and the authors concluded that acylamino and glycerol groups of the glycolipids were essential for the inhibitory activity on IAV multiplication [92]. From this new series, **204d** was the most potent, with an IC_50_ of 60.8 µM, and may provide a point of exploration of unique aminoglycoglycerolipids in drug discovery for pneumonia caused by viruses. In fact, this compound was selected for preliminary inhibitory studies on IAV infection *in vivo*. The results of this study showed that **204d** exhibited a significant reduction of viral titers in the lungs of IAV-infected mice at a dose of 5 mg/Kg/d, with a striking increase of the survival rate (90%) compared to the infected group treated with oseltamivir.

### 3.2. Crasserides and Isocrasserides

**Figure 20:**
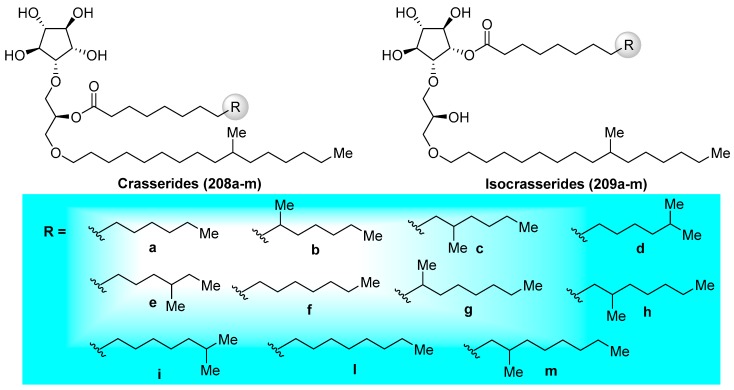
Structures of the crasserides (**208a**–**m**) and the isocrasserides (**209a**–**m**).

Crasserides (**208a**–**m**) (Figure 20), initially isolated by Fattorusso et al. [93] from the Caribbean sponge *Pseudoceratina crassa* (family Dysideidae), represent unique glycolipids that resemble common glycoglycerolipids by the replacement of an usual sugar by a five-membered cyclitol and the 1-*O*-acyl group present in the glycerol unit by an 1-*O*-alkyl group. Since their first isolation, the crasserides have been found in several families of sponges, including *Verongula gigantea* (family Dysideidae), *Aplysina fulva* (family Aplysinidae), *A. cauliformis* (family Aplysinidae), *Neofibularia nolitangere* (family Mycalidae), *Agelas clathrodes* (family Agelasidae), *A. dispar* (family Agelasidae), *A. conifera*, *A. longissima*, *Plakortis simplex* (family Plakinidae), *Ectyoplasia ferox* (family Raspailiidae), and *Siphonodictyon coralliphagum* (family Niphatidae) [94]. The structures of these interesting natural products were established and their stereochemistry was determined by exhaustive NMR spectroscopic analyses. Preliminary biological evaluations of these compounds revealed that the crasserides are potent feeding deterrents, with a concentration as low as 30 µg/cm^2^ of food pellets according to the antifeedant assay on the fish *Carassius auratus*. Almost ten years later, the same authors discovered from the same sponges a new family of related compounds, which are termed isocrasserides (**209a**–**m**) [94], which accompanied the crasserides in minor amounts, demonstrating that these compounds are genuine natural metabolites and not artifacts, as initially suspected. 

### 3.3. Myrmekiosides

**Scheme 18:**
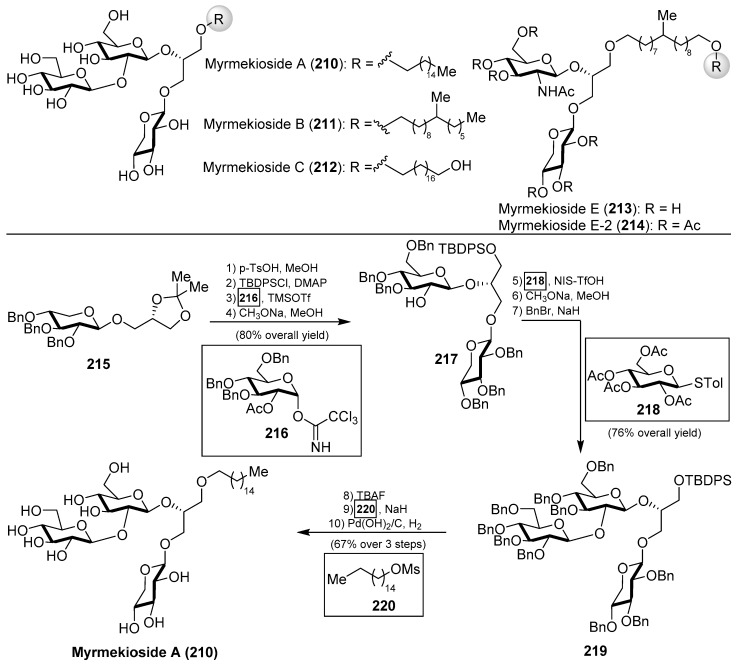
Structures of the myrmekiosides (**210**–**214**) and total synthesis of myrmekioside A (**210**).

The myrmekiosides (**210**–**212**) belong to the family of the glycoglycerolipids, isolated from the marine sponges *Myrmekioderma* sp. and *Trikentrion loeve* by Barnathan’s group and displayed potent antitumor activity [95]. In fact, their preliminary biological evaluations revealed that myrmekiosides altered the tumor cell morphology of H-ras transformed NIH3T3 fibroblasts to normal at concentrations of 5 µg/mL. Later, Barnathan et al. isolated a new and related glycoglycerolipid from *M. dendyi*, which was named myrmekioside E (**213**), and found that its peracetylated derivative (**214**) exerted significant cytotocixity against NSCLC-N6 and lung tumor A549 cells with IC_50_ values of 7.3 and 9.7 µM, respectively [96]. Like metabolites isolated from a marine origin, the scarcity of a bioactive material demanded its chemical synthesis as the only way to provide access in sufficient quantities for further biological evaluations. Prompted by this requirement, together with the unique mono-*O*-alkyl-diglycosylglycerol structure, Li’s group published in 2015 in what represents the first and, so far, only total synthesis of myrmekioside A (**210**) [97]. Accordingly, careful inspection of this unprecedented molecular framework led the authors to prepare the xylosylglycerol **215** as the starting material to incorporate in subsequent steps other sugar units. Thus, the glycerol moiety was elaborated for the next glycosylation reaction with the trichloroacetimidate **216** to obtain, in very high yield, diglycosylglycerol **217**, after removal of the acetate group under basic conditions. Compound **217** was then reacted with thioglycoside **218**, using NIS/TfOH as a promoter, to give the corresponding glycoglycerol in an excellent 90% yield. At this point, the authors had to replace the acetyl protecting groups by benzyl ethers, and then the resulting derivative **219** was driven to the completion of the synthesis of myrmekioside A (**210**), for which the etherification reaction to introduce the pending lipidic chain was carried out by reaction of the alcohol resulting from the desilylation process of **219** with the mesylate **220** (Scheme 18). This synthesis demonstrated the proposed structure for this natural product and, in addition, that the modular character of the synthetic strategy could be useful for the preparation of other myrmekioside derivatives and analogues for further biological studies.

### 3.4. Nigricanosides

**Figure 21:**
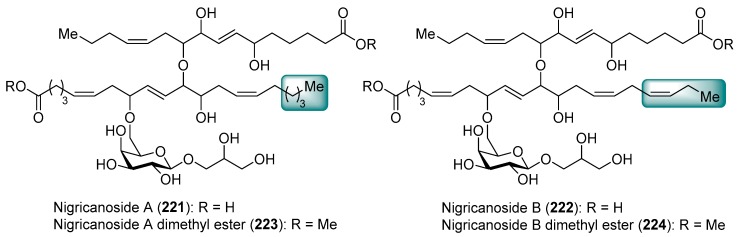
Structures of nigricanosides A (**221**), B (**222**), and their dimethyl esters, **223** and **224**.

Isolated as methyl esters (**223** and **224**) from the green alga *Avrainvillea nigricans* in Dominica [98], the nigricanosides (**221** and **222**) represent a unique and unprecedented class of glycolipids with outstanding antitumoral activity in the low nM range with an IC_50_ value of 3 nM for **221** against human breast cancer MCF-7 and colon cancer HCT-116 cell lines (Figure 21). More intriguing was the recognition of the ability of the nigricanosides to promote a polimerization of tubulins as the mechanism of action of their antiproliferative property. However, the tremendous scarcity of these natural products from the natural sources (Only 800 µg and 400 µg of the methyl esters of nigricanosides A and B, **223** and **224**, respectively, were obtained from 28 Kg of wet material!) represents a significant hurdle to gain further insight into the biological properties and to establish the absolute configuration of the compounds. Consequently, the total synthesis represents the only means to determine unambiguously the stereochemistry of the chiral centers and to have enough material for further biological screenings. 

**Scheme 19:**
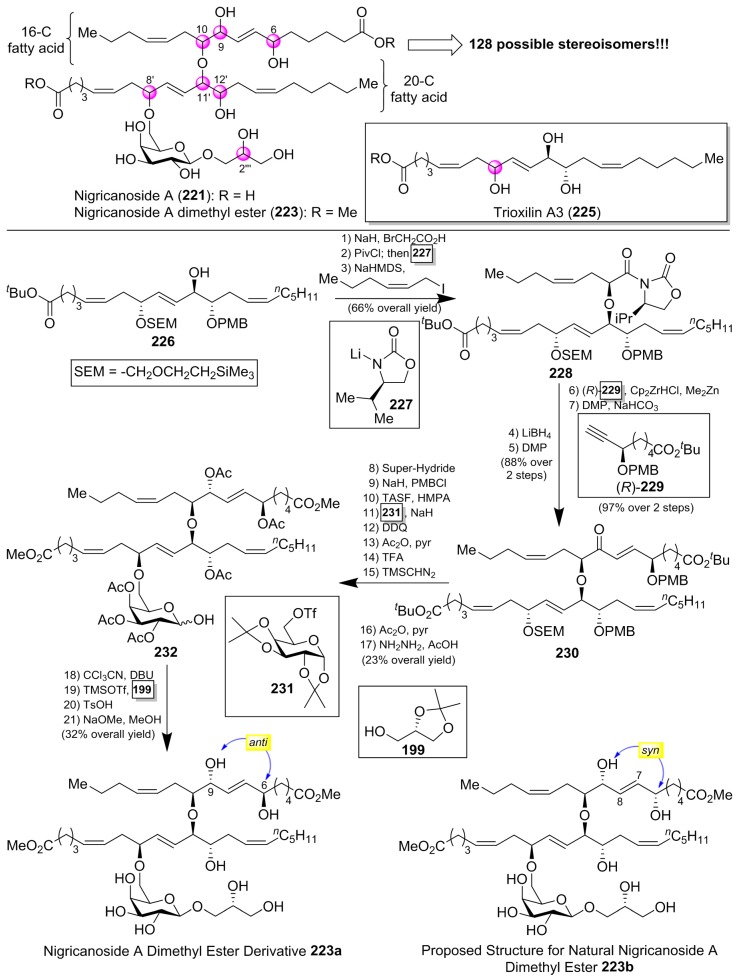
Synthesis of proposed natural nigricanoside A dimethyl ester (**223a**).

Thus, several synthetic approaches have been initiated for the lipidic fragments [99,100,101], but many of them have not solved the stereochemical assignment problem. In fact, this stereochemical assignment represents a formidable synthetic challenge because of the presence of seven chiral centers in the lipidic unit, which could provide up to 128 possible stereoisomers, assuming that the configuration for the sugar unit and the geometry of the double bonds were correctly established (Scheme 19). This synthetic challenge was taken on by the McMillan [102] and Ready [103] groups, which used a flexible synthetic strategy capable of delivering all the possible stereoisomers. In addition, the knowledge of the stereochemistry of a related 20-C fatty acid, trioxilin A3 (**225**), isolated and identified as a hydrolysis product of the natural epoxide hepoxilin A3, led the authors to propose this stereochemistry for the 20-C unit of the nigricanosides. Thus, according to Scheme 19, they prepared the ester **226** in a convergent and efficient way from (*R*)-glycidol as the chiral source, which was prepared for the subsequent assembly of the 16-C fatty acid unit via an ether linkage. To this aim, the free alcohol of **226** was treated with 2-bromo acetic acid and, after the incorporation of the chiral auxiliary **227**, the alkylation with (*Z*)-1-iodo-2-hexene afforded, with a complete stereoselectivity, the corresponding alkylated product **228**. The use of the enantiomer *ent*-**227** provided the corresponding stereoisomer at this position. By continuation of the product **228**, the elongation of the second fatty acid was achieved in four additional steps, in which the chiral ester (*R*)-**229** was incorporated via a hydrozirconation reaction with the Schwartz reagent with a subsequent transmetalation with dimethylzinc. In a similar manner, the authors used the enantiomer (*S*)-**229** to generate all the possible stereochemical combinations of the lipidic fragment. The completion of the lipidic moiety was undertaken in five additional steps, in which the resulting product **230** was prepared for the introduction of the sugar moiety. In this event, the alcohol derived from the deprotection of the SEM group was treated with the galactosyl triflate **231** to obtain glycolipid **232**, which was finally directed towards the nigricanoside A methyl ester via a glycosylation reaction of the trichloroacetimidate derivative of **232** with the alcohol **199**. Unfortunately, the resulting synthetic nigricanoside **223a** did not match with the reported natural product and, furthermore, was inactive against HCT-116 and MCF7 tumoral cell lines. A detailed comparative analysis of the ^1^H NMR spectra of synthetic diester **223a** and natural nigricanoside A dimethyl ester **223** revealed that the most important differences were located at the C7-C8 *trans* olefin region. These spectroscopic differences led the authors to propose a C6/C9 *syn* relationship for the natural product instead of the initially proposed *anti* relative configuration. 

Consequently, the nigricanoside derivative **223b** was prepared in the same way as for **223a**, and all its spectroscopic and physical properties matched with those reported for the natural product dimethyl ester. Despite this structural correspondence, it was surprising to find that neither **223b** nor its epimer at the glycerol subunit displayed cytotoxicity against HCT116 or MCF7 cells up to 10 µM. Due to the lack of cytotoxicity for the synthetic material compared to the reported activity for the natural product an explanation is warranted. One such explanation may be the presence of an unidentified product in the fractions corresponding to the natural nigrecanosides as the molecule responsible for the antitumoral activity exhibited by these natural products. The brilliant and outstanding synthetic work by McMillan and Ready’s groups allowed the stereochemical assignment of these fascinating natural products. However, the biological properties of the synthetic nigricanosides have opened an important uncertainty about the identity of the compound responsible for the antitumoral activity detected for the isolated compounds, which must be resolved in future investigations.

## 4. Chemistry and Biology of Atypical Glycolipids

### 4.1. Agminosides

**Figure 22:**
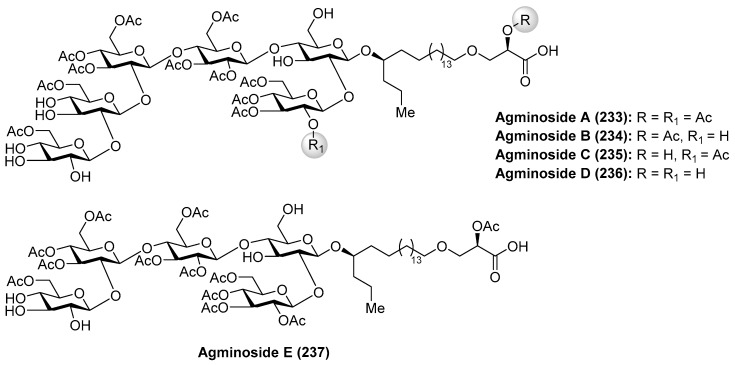
Structures of agminosides A–E (**233**–**237**).

Agminosides A–E (**233**–**237**) were isolated from the New Zealand sponge *Raspailia agminata* by Northcote et al. [104] (Figure 22). Structurally, these compounds possess only one type of aglycone, and contain up to six partially acetylated glucose residues that differ only in the level of acetylation and the number of sugars. Their structural similarity made their separation challenging, which was achieved only after repetitive normal-phase chromatography, which was more difficult due to the lack of a chromophore. Mass spectrometry-guided isolation and extensive NMR analysis, together with chemical derivatization, were used for the identification of their structures. 

### 4.2. Ancorinosides

**Figure 23:**
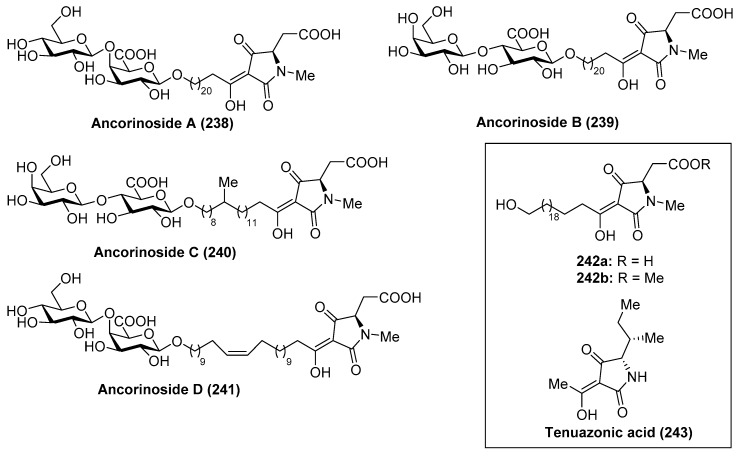
Structures of ancorinosides A–D (**238**–**241**).

Ancorinoside A (**238**) was isolated from the marine sponge *Ancorina* sp. by Ohta et al. [105] through bioassay-guided purification of the crude extract. This natural product is the first metabolite that possesses a tetramic acid ring derived from a d-amino acid, as well as a unique 21-methylene side chain. This compound was able to inhibit the blastulation of the starfish (*Asterina pectinifera*) embryo. Some years later, ancorinosides B–D (**239**–**241**) were isolated from the marine sponge *Penares sollasi* by Fusetani et al. [106] (Figure 23). Their structures were elucidated by spectroscopic and chemical methods, consisting of a tetramic acid glycoside related to ancorinoside A (**238**). In contrast to ancorinoside A (**238**), ancorinosides B–D (**239**–**241**) inhibited membrane type I matrix metalloproteinase (MT1-MMP) with IC_50_ values in the range of 180–500 µg/mL. An SAR study carried out by the same authors, wherein the aglycone of ancorinoside B (**242a**), its methyl ester **242b**, and tenuazonic acid (**243**) were tested, showed that **243** exhibited the best results, while **242a** and **242b** were slightly more potent than ancorinoside B (**239**). These results indicated that the two carboxylic acid groups were irrelevant for the biological profile and pointed out the importance of the tetramic acid group for this biological activity. In the same year, Ikegami et al. [107] isolated the magnesium salt of ancorinoside A (**238**) from the marine sponge *Ancorina* sp., which exhibited an inhibitory activity of the blastulation of the starfish *Asterina pectinifera* embryo similar to ancorinoside A (**238**). This result indicated that the presence of Mg^2+^ ions, which act as transmembrane transport and influence the fluidity and permeability of the membrane, did not affect the inhibitory activity of ancorinoside A (**238**).

**Scheme 20:**
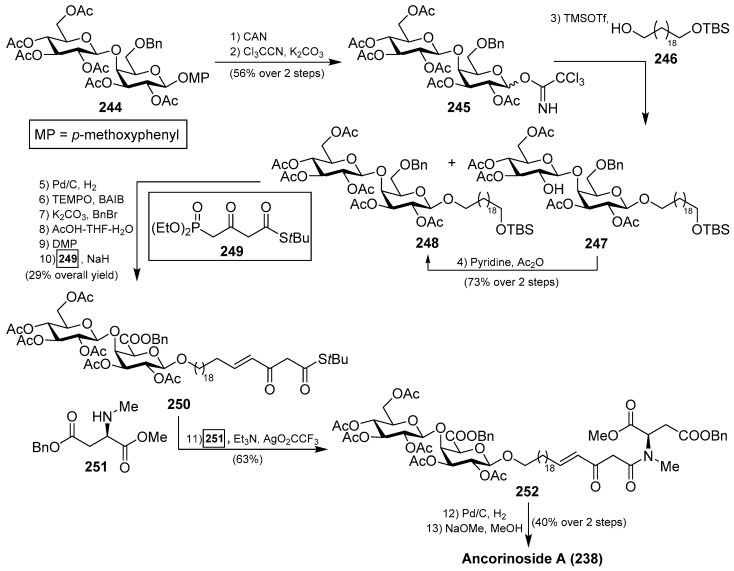
Total synthesis of ancorinoside A (**238**).

A total synthesis of ancorinoside A (**238**) was achieved in 1.6% yield in 18 steps by Schobert et al. [108], as described in Scheme 20. The key steps were the Schmidt glycosylation for the introduction of the lipidic spacer, a TEMPO oxidation to the uronic acid, functionalisation of the spacer terminus, and final Dieckmann cyclisation for the construction of the tetramic residue. Thus, the authors started the synthesis from peracetylated β-d-galactose, which was rapidly transformed into the disaccharide **244** in six steps in 49% overall yield. Oxidative deprotection, employing CAN, followed by generation of the corresponding imidate, afforded a separable α/β mixture of **245** in 56% yield over two steps. From **245**, the glycosylation reaction with a spacer precursor **246** was achieved by the use of TMSOTf as an activator to obtain a mixture of the desired disaccharide **248** together with deacetyl **247**, which was reacetylated to generate **248** in a 73% yield in two steps. Subsequent debenzylation reaction, TEMPO oxidation of the alcohol to the corresponding uronic acid, esterification of the acid, removal of the silyl group, followed by Dess–Martin periodinane oxidation to the corresponding aldehyde, and a final *E*-olefination with Ley’s β-ketophosphonate **249** afforded **250** in 29% overall yield. The coupling between **250** and **251** was achieved by the use of Ley’s protocol mediated by a silver salt to obtain the β-ketoamide **252** in 63% yield. Debenzylation of **252,** followed by a final base-induced Dieckmann cyclisation with concomitant deacetylation, afforded ancorinoside A (**238**) (Scheme 20).

### 4.3. Bartolosides

**Figure 24:**
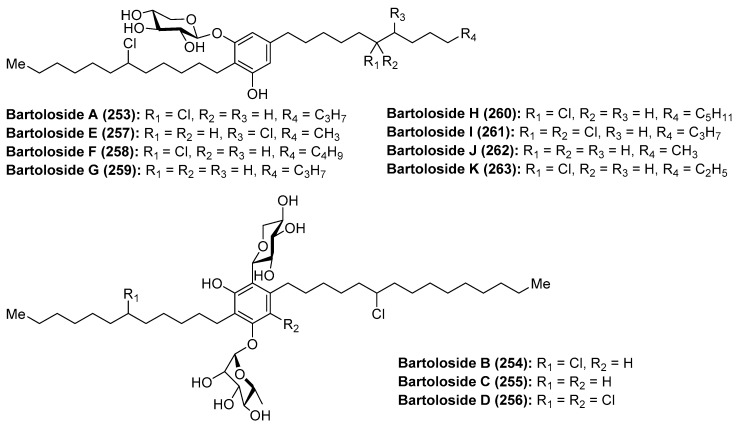
Structures of bartolosides A–K (**253**–**263**).

Bartoloside A (**253**) was isolated from the cyanobacterium *Nodosilinea* sp. LEGE 06102 [109] and bartolosides B–D (**254**–**256)** were isolated from the cyanobacterium *Synechocystis salina* LEGE 06155 through a bioassay-guided fractionation of the crude extract by Balskus et al. (Figure 24). A year later, Leao et al. [110] isolated bartolosides E–K (**257**–**263**) from the strain LEGE 06099 of *S. salina*. 

Structurally, these glycolipids from marine cyanobacteria possess unique structures featured by the presence of a dialkyl-resorcinol core, decorated by one or two xylose units with chlorine groups in the aliphatic chains and a C-glycosyl moiety in the case of bartolosides B (**254**), C (**255**), and D (**256**). In the case of bartolosides E–K (**257**–**263**), these can be considered as analogues of bartoloside A (**253**) that differ in the alkyl chain lengths or halogenation patterns.

The chlorinated dialkylresorcinol core of the bartolosides conferred a challenge for their structural elucidation, which was finally established by combined biosynthetic and bioinformatic analysis. With regards to their biological features, the bartolosides are not known to possess strong biological activities, but their high abundance inside the cells suggests that they may have an important biological role that has to be investigated.

### 4.4. Caminosides

**Figure 25:**
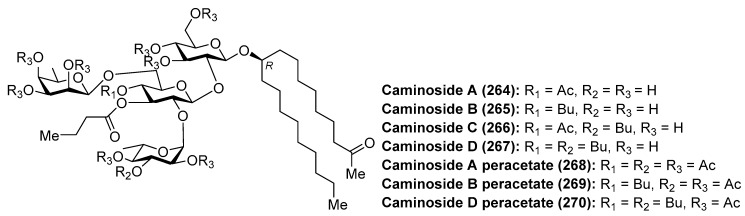
Structures of caminosides A–D (**264**–**267**) and their peracetylated derivatives (**268**–**270**).

The caminosides (**264**–**270**) (Figure 25) are glycolipids with interesting antimicrobial activity isolated from the marine sponge *Caminus sphareoconia* by Andersen et al. [111]. Firstly, caminoside A (**264**) was discovered after a biological screening of crude extracts of the marine sponge for its ability to inhibit the secretion of *Escherichia coli*-secreted proteins (Esps) by enteropathogenic *E. coli* (EPEC), a major cause of infantile diarrhea that actually represents the main cause of mortality of children in developing countries. Interestingly, pathogenic *E. coli*, and not nonpathogenic *E. coli*, employed the named type III secretory apparatus to deliver Esps. Thus, compounds capable of inhibiting the type III secretion system would affect the pathogenic *E. coli* and not the comensal *E. coli* flora. Actually, the inhibition of the type III secretion system produced the attenuation of the pathogenicity without killing the bacteria. Thus, caminoside A (**264**) displayed an IC_50_ of 20 µM, not inhibiting the growth of Gram-negative bacteria such as *E. coli* (MIC > 100 µg/mL). In addition, caminoside A (**264**) showed significant antibacterial activity against methicillin-resistant *Staphylococcus aureus* (MIC 12 µg/mL) and vancomycin-resistant *Enterococcus* (MIC 12 µg/mL). Some years later, the same authors isolated and identified new members of the caminosides (B–D, **265**–**267**) with similar biological activities [112]. In all the cases, the authors prepared the peracetylated derivatives **268**–**270** to facilitate their separation and structural determination. On the other hand, the unusual structure of these glycolipids is featured by the presence of a nonglycerol lipidic chain, whose stereochemistry at C-10 position was recently determined by circular dichroism [113]. 

**Scheme 21:**
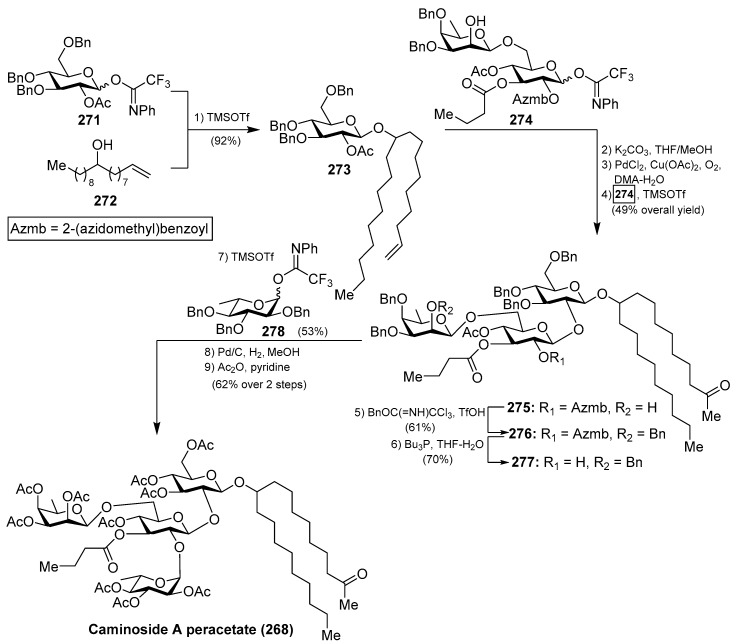
Total synthesis of caminoside A peracetate (**268**).

**Scheme 22:**
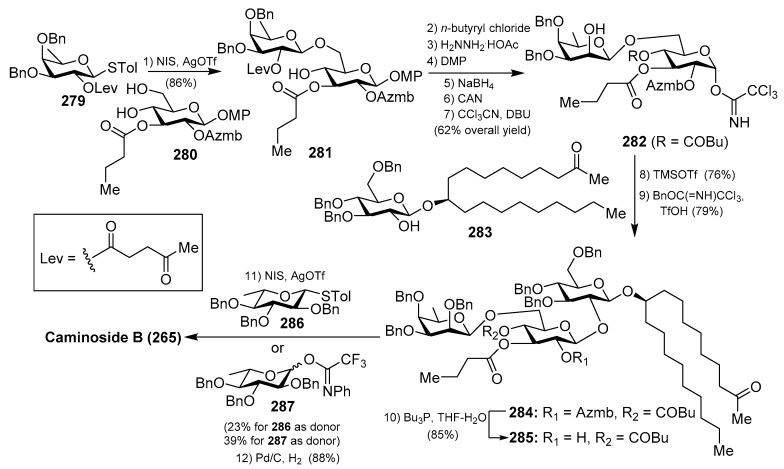
Total synthesis of caminoside B (**265**).

The first synthesis of caminoside A (**264**) was reported by Yu et al. [114], wherein they succeeded in sorting out the different issues concerning the stereochemistry of the glycosidic bonds, particularly the 1,2-*cis*-β-mannopyranoside-type linkage of the 6-deoxy-talose unit. This issue was solved by the use of a 2-*O*-Lev (levulinyl) fucosyl derivative to obtain stereoselectively the β-glycosidic linkage with a glucopyranosyl derivative, and then an inversion of the C-2 configuration via oxidation/reduction of the alcohol. Having constructed the disaccharide **274**, its assembly with the glucosyl derivative **273**, prepared via glycosylation of the trichloroacetimidate **271** and alcohol **272**, was undertaken after removal of the acetate and Wacker oxidation of **273**. The resulting trisaccharide **275** was then prepared for the introduction of the 6-deoxy-l-glucosyl unit, for which **275** was benzylated at the 2-OH group of the deoxy-talose fragment, followed by the selective removal of the 2-(azidomethyl)benzoyl group (Azmb) that was possible in the presence of the acetate and butyrate groups by treatment with tributylphosphine. With the resulting acceptor **277** in hand, the reaction with the donor **278**, under the action of catalytic TMSOTf, provided the corresponding caminoside derivative in 53% yield, exclusively as the α-anomer, which was finally subjected to a global deprotection step and acetylation to obtain caminoside A peracetate (**268**) (Scheme 21). More recently, Li et al. have reported the total synthesis of caminoside B (**265**) in a closely related strategy, as summarized in Scheme 22, through key intermediates **282**–**285** and by efficient and stereoselective glycosylation reactions [115]. 

### 4.5. Clathrosides and Isoclathrosides

**Figure 26:**
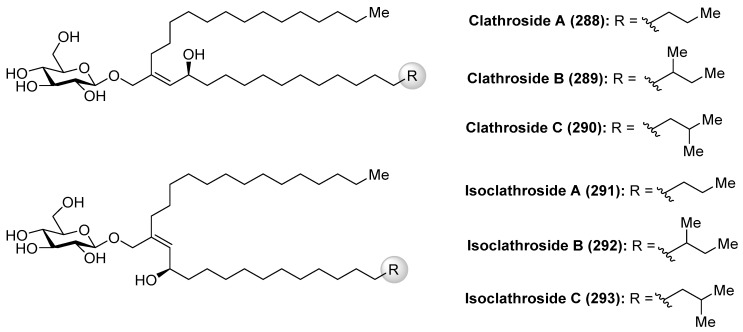
Structures of clathrosides A–C (**288**–**290**) and isoclathrosides A–C (**291**–**293**).

Clathrosides A–C (**288**–**290**) and isoclathrosides A–C (**291**–**293**) (Figure 26), isolated from the Caribbean sponge *Agelas clathrodes* by Mangoni et al. [116], are glycosides containing a very long chain alcohol derived from fatty acids. The structural differences between the clathrosides and the isoclathrosides can be found in the configuration and in the branching of the alkyl chains. Stereostructures of the clathrosides were elucidated by NMR and CD spectroscopy, mass spectrometry, and chemical degradation studies. Clathroside A (**288**) and isoclathroside A (**291**) did not show significant activity on the immune system of mammals despite the structural similarity between clathrosides and the immunoactive simplexides, which will be described in the following section.

### 4.6. Discoside

**Figure 27:**
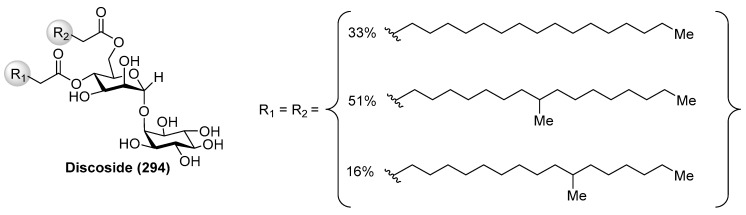
Structure of discoside (**294**).

Discoside (**294**) (Figure 27) was isolated as a mixture of homologues from the marine sponge *Discodermia dissoluta* by Fattorusso et al. [117]. The complete stereostructure of discoside was determined by mass spectrometric and NMR spectroscopic techniques, together with CD analysis of degradation products. Interestingly, discoside (**294**) represents the first reported glycolipid with a 4,6-*O*-diacylated α-linked to the 2-hydroxyl group of a *myo*-inositol unit [2-*O*-(4,6-di-*O*-acyl-α-d-mannopyranosyl)-*myo*-inositol]. The *myo*-inositol mannoside is a well-known building block of phosphatidylinositol mannosides and of their multiglycosylated form, lipomannans, which exhibit immunoregulatory effects. However, such types of compounds have never been reported from marine sponges, 1-*O*-pentadecanoyl-2-*O*-(6-*O*-heptadecanoyl-α-d-mannopyranosyl)-*myo*-inositol being the only closely discoside-type compound, which was isolated from various strains of *Propionibacterium*. This finding suggests that discoside is synthesized by symbiotic cyanobacteria associated with the marine sponge.

**Scheme 23:**
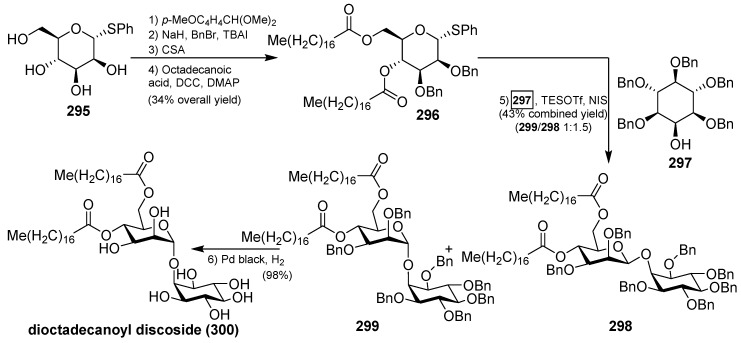
Total synthesis of dioctadecanoyl discoside **300**.

The synthesis of 4,6-di-*O*-octadecanoyl discoside (**300**) was recently accomplished by Florence et al., starting with the preparation of the thiomannoside donor **296**. From α-d-phenylthiomannoside **295**, sequential protection of the 4- and 6-OH, benzylation of the remaining two hydroxyl groups, acidic methanolysis of the anisylidene acetal, followed by esterification under Steglich conditions, provided the donor **296** in 34% overall yield. The key glycosylation reaction between the donor **296** and the acceptor **297**, which was synthesized in six steps from a readily available orthoformate derivative of *myo*-inositol, was achieved with the activation of **296** using NIS and TESOTf, followed by the reaction with **297** to give a mixture of separable anomers **298** and **299** in 43% yield (α:β ratio 1:1.5). The total synthesis was completed by final debenzylation of the minor α isomer **299** to provide 4,6-di-*O*-octadecanoyl discoside (**300**) in a 98% yield (Scheme 23) [118].

### 4.7. Erylusamides

Erylusamides A–D (**301**–**304**) were isolated from the marine sponge *Erylus cf. deficiens* by Santos et al. through a bioassay-guided fractionation in order to find inhibitors of indoleamine 2,3-dioxygenase (IDO) [119]. The structures of erylusamides were established by NMR spectroscopy, HREIMS and chemical derivatization. These compounds possess a pentasaccharide moiety with unusual highly acetylated d-glucose, together with d-xylose and d-galactose units, and unprecedented aglycones, which are structurally featured by long chains of dihydroxyketo amides.

**Figure 28:**
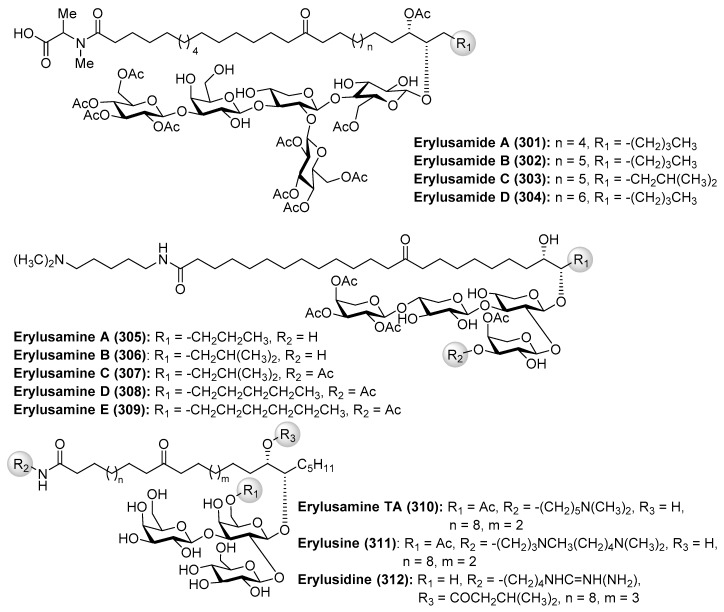
Structures of erylusamides A–D (**301**–**304**) and related glycolipids (**305**–**312**).

On the other hand, the related erylusamines A–E (**305**–**309**) were isolated from the marine sponge *Erylus placenta* by Fusetani et al. [120,121]. These compounds are interleukin-6 (IL-6) receptor antagonists. Erylusamine TA (**310**), erylusine (**311**), and erylusidine (**312**) (Figure 28) were isolated from *Erylus cf. lendenfeldi* by Kashman et al. [122]. These compounds were not tested for any biological activities due to the fact that they were not isolated as pure compounds but together with a small amount of other homologues.

### 4.8. Ieodoglucomides and Ieodoglycolipid

**Figure 29:**
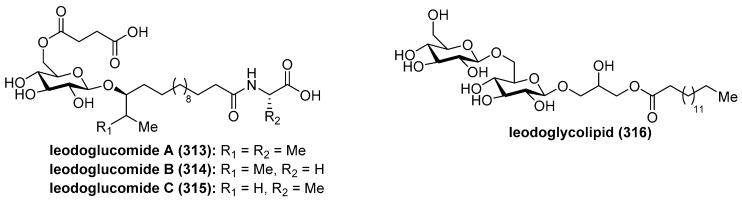
Structures of ieodoglucomides A–C (**313**–**314**) and ieodoglycolipid (**316**).

Ieodoglucomides A (**313**) and B (**314**) (Figure 29) were isolated from a marine-derived bacterium, *Bacillus licheniformis,* in 2012 by Shin et al. [123] and their structures were elucidated by a combination of NMR spectroscopic analysis of the natural products with the spectroscopic and physical analyses of the products derived from their acid hydrolysis. Iedoglucomides are unique glycolipopeptides that contain an amino acid (l-ala for ieodoglucomide A and glycine for iedoglucomide B), an unprecedented fatty acid (14-hydroxy-15-methylhexadecanoic acid), succinic acid, and a sugar (β-d-glucose). Iedoglucomide B (**314**) showed moderate antimicrobial activity against Gram-positive and Gram-negative bacteria, antifungal as well as growth inhibition against lung cancer (NCI-H23) and stomach cancer (NUGC-3) cell lines with GI_50_ values of 25.18 and 17.78 µg/mL, respectively.

Ieodoglucomide C (**315**) and ieodoglycolipid (**316**) were isolated from the fermentation broth of the marine-derived bacterium *Bacillus licheniformis* [124]. In contrast to iedoglucomide B (**314**), these compounds showed good antibiotic properties against *Staphylococcus aureus*, *Bacillus subtilis*, *B. cereus*, *Salmonella typhi*, *Escherichia coli*, and *Pseudomonas aeruginosa* with MIC values in the 0.01–0.05 µM range. In addition, these compounds inhibit the mycelial growth of plant pathogenic fungi *Aspergillus niger*, *Rhizoctonia solani*, *Botrytis cinerea*, and *Colletotrichum acutatum* as well as the human pathogen *Candida albicans,* with MICs values of 0.03–0.05 µM. The antimicrobial profiles of ieodoglucomide C (**315**) and ieodoglycolipid (**316**) suggest that they could be potential bioprobes for the development of useful antibiotics and fungicides.

**Scheme 24:**
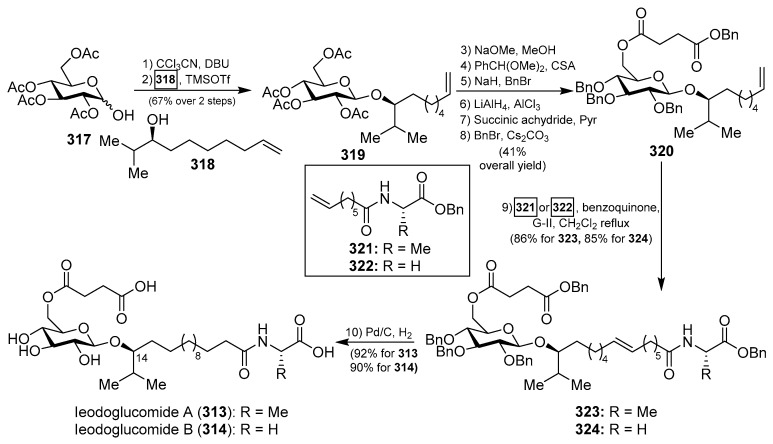
Total synthesis of ieodoglucomides A (**313**) and B (**314**).

Total syntheses of ieodoglucomide A (**313**) and B (**314**) were carried out by Reddy et al. [125] starting from d-glucose and using β-glycosylation and olefin cross-methatesis reactions as the key steps. Thus, the synthesis started with the conversion of **317**, prepared from d-glucose in two steps, into the corresponding α-trichloroacetimidate, which was employed in the key glycosylation reaction, promoted by TMSOTf, with the alcohol **318** to obtain the β-glycoside **319** in 67% yield in two steps. The introduction of the succinyl group onto the sugar to generate **320** was accomplished in six steps in 41% overall yield from **319**. Next, the key olefin cross-metathesis between **320** and **321** or **322**, under the action of the Grubbs 2nd generation catalyst, gave the protected glycolipids **323** and **324** in excellent 86% and 85% yields, respectively. Final hydrogenation provided ieodoglucomides A (**313**) and B (**314**) in 92 and 90% yields, respectively (Scheme 24).

**Scheme 25:**
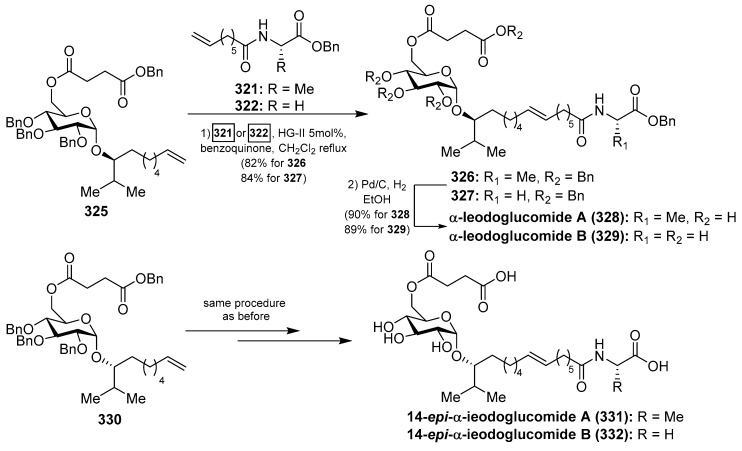
Total synthesis of α-isomers of ieodoglucomide A (**328**) and B (**329**) and their α-14-*epi* analogues (**331** and **332**).

In order to identify analogs of ieodoglucomides with improved biological properties, the same authors prepared the α-isomers of ieodoglucomides A and B (**328** and **329**) and their C-14-epimers (**331** and **332**) (Scheme 25) [126]. As described in Scheme 25, the syntheses of these analogues were accomplished according to the same synthetic strategy employed for the synthesis of the natural products, starting from the α-anomer **325** for the synthesis of the analogues **328** and **329**, and from the epimer **330** for the syntheses of the analogues **331** and **332**. These stereochemical analogues, together with the natural ieodoglucomides, were tested for cytotoxicity against various cancer cell lines and found that ieodoglucomide A (**313**) and B (**314**), as well as their α-isomers (**328** and **329**), did not exhibit cytotoxicity in any cell lines tested. However, their α-C14 epimers (**331** and **332**) showed an inhibition of proliferation of DU145 and HeLa cells with IC_50_ values in the range of 15.2–35.5 µM. In addition, this biological study demonstrated that the activity of caspases-3 and -9 increased when these cell lines were treated with these compounds **331** and **332**, suggesting that the α-*epi* isomers of the ieodoglucomides led to the activation of pathway-initiating apoptosis in DU145 and HeLa cells.

### 4.9. Pachymoside A

**Figure 30:**
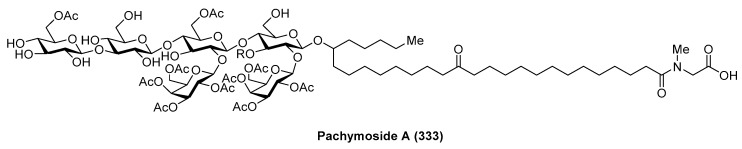
Structure of pachymoside A (**333**).

Pachymoside A (**333**) (Figure 30) was isolated from the North Sea marine sponge *Pachymatisma johnstonia* through a bioassay-guided fractionation in order to find inhibitors of the bacterial type III secretion system (TTSS), carried out by Andersen et al. [127]. Its structure was elucidated by NMR spectral analysis and chemical degradation. This structural study revealed that pachymoside A (**333**) possesses complete acetylation of the galactose residues (the eight galactose hydroxyls are acetylated) and only partial acetylation of the glucose residues (the C-6 hydroxyls of β-glucose and d-glucose are acetylated). Despite its promising activity as a TTSS inhibitor, pachymoside A (**333**) is not a true TTSS inhibitor. It seems that the biological activity exhibited is due to its ability to activate extracelular bacterial proteases that rapidly degrade the excreted Esps, resulting in a false indication of inhibition.

### 4.10. Plaxyloside

**Figure 31:**
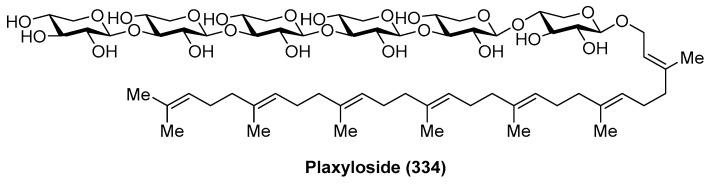
Structure of plaxyloside (**334**).

Plaxyloside (**334**) (Figure 31) was isolated as its peracetate from the Caribbean sponge *Plakortis simplex* by Fattorusso et al. [128]. Its structure was established by NMR spectral analysis and chemical methods. Plaxyloside (**334**) is a glycolipid with a long linear polyisoprenoid alcohol aglycone and a linear carbohydrate chain of β-xylopyranose units and represents the first natural oligosaccharide that contains more than three (1→3) linked xylopyranosides. The biological activity of this compound was not evaluated.

### 4.11. Roselipins

**Figure 32:**
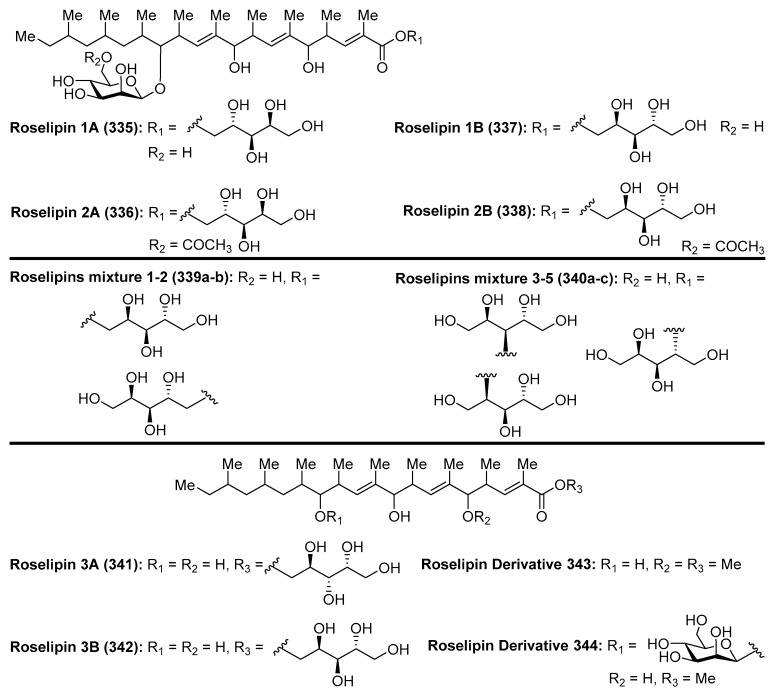
Structures of the roselipins (**335**–**340**) and derivatives **341**–**344**.

Roselipins (**335**–**338**) (Figure 32) were isolated from the culture broth of fungus *Gliocladium roseum* KF-1040 by Omura et al. [129,130,131]. Later, a mixture of five new roselipins derivatives, separated in two fractions, named roselipins mixture 1–2 (**339a**–**b**) and mixture 3–5 (**340a**–**c**), were isolated from an extract of the fungus *Clonostachys candelabrum* by Singh et al. [132]. These compounds differ by the position of esterification of the arabinitol residue. Overall, the roselipins have a unique structure featured by a highly methylated C-20 fatty acid skeleton modified with d-mannose and d-arabinitol residues. Extensive biological evaluations of the roselipins revealed that these compounds displayed a broad range of biological activities. In particular, these compounds inhibit diacylglycerol acyltransferase (DGAT) with IC_50_ values ranging from 15 to 22 µM in an enzyme assay system using rat-liver microsomes, showing that are selective inhibitors of DGAT2 (IC_50_ = 30–50 µM) [133]. An SAR study carried out by Omura et al. [134] revealed that demannosyl roselipins 3A (**341**) and 3B (**342**), prepared from the natural products via enzymatic degradation, conserved DGAT inhibitory activity, in contrast to the dearabinitoyl derivatives (**343** and **344**), which completely lost their activity, suggesting that the arabinitoyl fatty acid core is essential for eliciting DGAT inhibitory activity. In addition, the roselipin derivatives 3A (**341**) and 3B (**342**) were more potent in the cell assay than the roselipins **335**–**338**, indicating that these derivatives are more membrane-permeable than natural roselipins. These compounds also exhibited antimicrobial activity against *Saccharomyces cervisiae* and *Aspergillus niger* and showed a cytotoxic effect on Raji cells at 39 µM. Furthermore, the mixture of roselipins 2A and 2B (**336** and **338**) inhibited HIV-1 integrase with IC_50_ = 8.5 µM [135]. On the other hand, Ondeyka et al. [136] reported that roselipins 2A (**336**), 2B (**338**), and 1A (**335**) blocked the CXCR3 receptor interaction of IP-10 ligand with IC_50_ = 14.6, 23.5, and 41 µM. The roselipins were also identified as anthelmintic compounds [133].

### 4.12. Simplexides

**Scheme 26:**
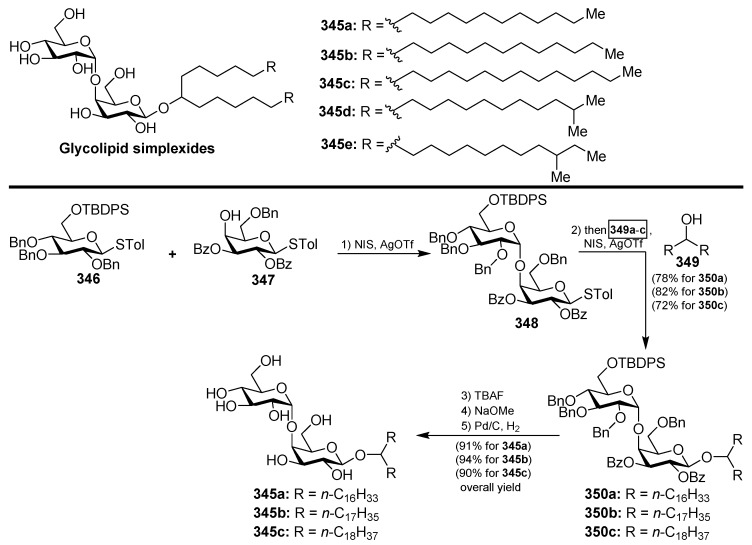
Structures and synthesis of the simplexides (**345a**–**c**).

The same marine sponge that produces the plakosides and plaxyloside (Sections Plakosides and Plaxyloside), *Plakortis simplex*, has provided other types of glycolipids with immunosuppressive properties, the simplexides (**345a**–**e**), which were isolated as a mixture of related disaccharides [137]. Even though these compounds could not be separated by HPLC techniques, the authors were able to establish their structures via degradation and subsequent CG-MS analyses of the resulting methyl esters of the lipidic chains. Despite the remarkable structural differences with the plakosides, the simplexides share a very similar biological profile, showing potent inhibitory activity of the proliferarion of T cells in the murine immune system when stimulated with concanavalin A through a noncytotoxic mechanism. Further biological studies demonstrated that the immunosuppressive effects of the simplexides are due to the induction that they exert on the expression and release of cytokines and chemokines from human monocytes by direct interaction with the CD1d receptors, expressed in these cells [138]. In addition, the simplexides induce the expansion of *i*NKT (natural killer T cells with an invariant T cell receptor alpha chain). An interesting synthesis for the simplexides was recently developed by Yingxia et al. [139], in which thioglycosides were employed to construct the complete simplexide scaffold in a one-pot glycosylation method. Thus, the armed donor **346** reacted with the disarmed acceptor **347** by the action of the promoter system NIS/AgOTf to obtain, with a high α-selectivity, the disaccharide **348**. Without isolation of this product, the corresponding alcohol **349** was added to the crude mixture with additional promoters (NIS/AgOTf) to proceed with a second glycosylation reaction to provide **350a**–**c**, which were finally isolated in excellent yields and stereoselectivity. Final deprotection of **350a**–**c** gave simplexides **345a**–**c** (Scheme 26). 

**Scheme 27:**
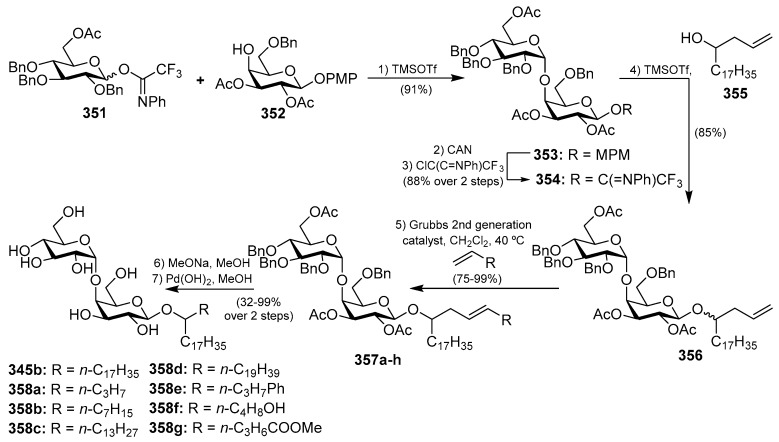
Synthesis of the natural simplexide **345b** and analogues **358a**–**g**.

More recently, a synthesis of this class of glycolipids by Yu et al. [140] exploited the cross-metathesis reaction to build the lipidic chain from the allyl disaccharide **356**, efficiently prepared from glycosyl derivatives **351** and **352** via trichloroacetimidates. The crucial cross-metathesis reaction was achieved by the action of the Grubbs 2^nd^ generation catalyst to obtain simplexides **357a**–**h** in very good yields, which were transformed into the final simplexides **345b** and **358a**–**g** in two additional steps (Scheme 27). This new efficient and concise synthesis of the simplexides and analogues will allow the determination of the structural factors that govern their interesting biological activities, a study that has yet to be completed by the authors.

## 5. Conclusions

Carbohydrates are often found as primary metabolites in the form of monomers, oligomers, or polymers, and play essential functions for life. However, in the realm of natural products, carbohydrates can be found mainly as components of glycoconjugates. In this case, they play important roles in conferring certain physical, chemical, and biological properties to the carrier molecules, being essential in cellular-recognition processes. A relevant example of the glycoconjugates are glycolipids and, notably, the glycolipids from a marine origin, which represent an important class of natural products with wide structural diversity and a broad range of biological activities, including antitumoral, antibiotic, antiviral, antimalarial, immunostimulatory, and neuritogenic activities. However, their difficult accessibility from natural sources, coupled with their extreme scarcity, has made their chemical synthesis essential to provide their access for additional studies. In addition, further understanding of their mechanisms of biological action allows for the rational design and synthesis of new analogues. Through the present review, we have presented the molecular and biological diversity of the glycoclipids derived from marine sources, giving special emphasis on their syntheses as an important tool to confirm their molecular structures, gain insight into their biological activities, and the design of analogues for the development of new drugs. In conclusion, the readers have hopefully realized a greater interest in this class of natural products and the awesome power of chemical synthesis for the development of valuable bioactive compounds based on these natural products.
